# Recent Progress of CircRNAs in Hematological Malignancies

**DOI:** 10.7150/ijms.98156

**Published:** 2024-09-30

**Authors:** Ya-Bin Cui, Li-Jie Wang, Jin-Hui Xu, Hui-Jie Nan, Pei-Yao Yang, Jun-Wei Niu, Ming-Yue Shi, Yan-Liang Bai

**Affiliations:** 1 Department of Hematology, Henan University People's Hospital and Henan Provincial People's Hospital, Zhengzhou 450003, P.R. China.; 2 Department of Hematology, Zhengzhou University People's Hospital and Henan Provincial People's Hospital, Zhengzhou 450003, P.R. China.

**Keywords:** circRNA, hematological malignancies, resistance, leukemia, lymphoma, multiple myeloma

## Abstract

Circular RNAs (circRNAs) are now recognized as key regulators in the epigenetic control of genetic expression, being involved in a wide range of cellular activities such as proliferation, differentiation, and apoptosis. Their unique closed-loop structure endows them with stability and resistance to exonuclease degradation, making them not only key regulatory molecules within the cell but also promising biomarkers for disease diagnosis and prognosis, particularly in hematological malignancies. This review comprehensively explores the role of circRNAs in the pathogenesis, progression, and therapeutic resistance of common hematological malignancies. Furthermore, the review delves into the prognostic significance of circRNAs, underscoring their potential in predicting disease outcomes and treatment response. Given their extensive involvement in cancer biology, circRNAs present a frontier for novel therapeutic strategies.

## Introduction

In the 1970s, circular RNAs (circRNAs) were first identified in viruses as a closed circular non-coding RNA lacking a 5′-cap and a 3′-poly A tail^1^. Initially, circRNAs were regarded as mere products resulting from abnormal RNA splicing and did not garner significant interest from researchers in the subsequent years^2^, ^3^. CircRNAs are conserved, stable and specific to the developmental stages and tissues^4^, ^5^. Compared to linear RNAs, circRNAs have been shown to exhibit a high level of conservation at the nucleotide level across various eukaryotic organisms, ranging from yeast to humans^4^. Because of their inherent resistance to exonucleases, circRNAs seem to be highly stable transcripts with half-lives exceeding 24 to 48 hours^6^. CircRNAs may be easily detected since they were widely distributed in human peripheral blood and tissues^7^, ^8^. In recent years, numerous circRNAs have been detected in various eukaryotes through the implementation of molecular biotechnology methods, including high-throughput sequencing, microarray, and bioinformatics analysis^9^, and their expression has been identified to be cell-, tissue-, and timing-specific^10^, ^11^. Currently, a large number of studies have confirmed that circRNAs have regulatory functions in growth^12^-^14^, aging^15^, and disease^16^, especially in the occurrence and development of tumors^17^, ^18^. Studies have demonstrated that circRNAs significantly contribute to the progression of various cancers by acting as either tumor suppressors or oncogenes. They were being recognized as novel markers for the diagnoses and prognostic assessments^19^. In addition, circRNAs were involved in a range of biological and physiological functions, including cell proliferation, cancer spread, stem cell characteristics, the tumor environment, and evading the immune system^20^, ^21^, suggesting potential contributions to the pathogenesis of several human diseases. So far, circRNAs have been shown to have varied expression in cancerous tissues and play a role in the development of different types of tumors, such as hematological malignancies^22^. Meanwhile, studies has been established that circRNAs are involved in various pathological and physiological processes of blood cells, such as blood cell differentiation, proliferation, and apoptosis, as well as in the development and prognosis of hematological malignancies^23^.

This review comprehensively synthesizes both recent and prior research on circRNAs in hematological malignancies. It provides a detailed categorization of the roles circRNAs play in basic experimental settings (*in vivo* and *in vitro*) and in clinical practice. The review explores various aspects of the relationship between circRNAs and hematological malignancies, such as potential pathogenesis, their influence on cellular activities, associations with chemotherapy resistance, linkages with specific genes, and their value in clinical prognosis. Additionally, the review includes a focused summary of circRNAs in specific types of lymphomas, potentially offering new avenues for future research in this field.

## CircRNAS and Acute myeloid leukemia

Acute myeloid leukemia (AML) is a genetically diverse disease, and the degree to which cytogenetic and molecular features determine severity and impact treatment choices is a rapidly developing area of study^24^. A study indicated that the promotion of leukemia development by hsa_circ_0009581 was mediated through hsa-miR-150-5p, while hsa_circ_0001947 contributed to leukemia progression via hsa-miR-454-3p. These findings may offer novel insights into the pathogenesis of AML^25^. However, the pathogenesis of AML is still unclear, and recent studies on circRNAs have mainly focused on the regulation of leukemia cell proliferation, apoptosis, etc., as well as therapeutic resistance and prognosis.

### CircRNAS were highly expressed in AML

#### CircRNAs may paly a role of AML cells activities

M2-like macrophage polarization was reported to be induced by the AML cell-derived exosome circ_001264, which also regulated ras-associated factor 1 (RAF1) expression and activated the p38-STAT3 signaling pathway. By secreting programmed death-ligand 1 (PD-L1), polarized M2-like macrophages can cause PD-L1 upregulation. Therefore, researchers used anti-programmed cell death receptor ligand 1 (aPD-L1) in combination with exosome circ_001264 siRNA for *in vitro* antitumor therapy and found that the combination of exosome circ_001264 siRNA and aPD-L1 in a mouse model significantly reduced the leukemia tumor load, demonstrating a clear anticancer effect^26^. The expression of circZBTB46 was markedly elevated in AML patients and AML cells. Silencing circZBTB46 led to cell cycle arrest and decreased AML cell proliferation. Crucially, in these cells, the reduction of circZBTB46 significantly enhanced ferroptosis^27^. In AML patients and cells, circPLXNB2 was substantially expressed and exhibited greater stability compared to linear PLXNB2. The progression of the cell cycle, as well as apoptosis and proliferation of AML cells, was impacted by circPLXNB2 knockdown^28^. The levels of Zinc finger and BTB domain containing 20 (ZBTB20) and circ-SFMBT2 were higher in cells from AML patients. In AML cells, depletion of circ-SFMBT2/ZBTB20 induced apoptosis and hindered proliferation, migration, invasion, and glycolysis^29^. Peripheral blood samples and cells from AML patients have elevated levels of circPTK2. CircPTK2 knockdown increased cell death and induced cell cycle arrest while inhibiting AML cell growth and glycolysis^30^. Meanwhile, a study discovered that circ-PTK2 was substantially expressed in AML, inhibiting apoptosis and promoting AML cell proliferation through its targeting of the miR-330-5p/ FOXM1 axis^31^. In AML cell lines and bone marrow samples from children with AML, there was a rise in the expression of circ_KCNQ5. Circ_KCNQ5 knockdown significantly inhibited AML cell proliferation and promoted cell apoptosis^32^. The blood of juvenile AML patients and AML cells lines (HL-60 and NB4) showed significant expression of circ 0005774. Blocking circ_0005774 and/or overexpressing miR-192-5p in HL-60 and NB4 cells increased the rate of apoptosis, decreased cell viability, and reduced cell cycle entry. Additionally, it led to a decrease in proliferation markers such as proliferating cell nuclear antigen (PCNA), Cyclin D1, and B cell lymphoma 2 (Bcl-2)^33^. Circ_0094100 was elevated in AML tissues and cells. Circ_0094100 knockdown further decreased the rapamycin-mediated reduction in AML cell viability and cell cycle progression, while also increasing apoptosis. During the investigation of the underlying mechanism, circ_0094100 was found to act as a sponge for miR-217. Inhibition of miR-217 reversed the effects of circ_0094100 knockdown on the malignant behaviors of rapamycin-treated AML cells^34^. Tumor formation was observed *in vivo* experiments after overexpression of circRNA-DLEU2, which aided in the growth of AML cells and prevented their apoptosis^35^. Mature miR-34a expression levels in AML cells were downregulated by circ_POLA2 overexpression, but the expression levels of the miR-34a precursor were not affected. The results of the examination of cell proliferation indicated that miR-34a overexpression mitigated the effects of circ_POLA2 overexpression^36^. In AML cells, overexpression of circ-ATAD1 resulted in reduced production of miR-34b and increased methylation of the miR-34b gene. Additionally, circ-ATAD1 overexpression boosted AML cell proliferation, whereas miR-34b overexpression reduced cell proliferation. Furthermore, miR-34b overexpression decreased the proliferative effect caused by circ-ATAD1 overexpression^37^. AML cells have been shown to produce hsa_circ_0079480 at high levels. Loss-of-function tests have demonstrated that reducing hsa_circ_0079480 expression inhibited AML cell proliferation, and induced apoptosis *in vitro* through the miR-654-3p/HDGF axis^38^.

This section described the impact of four circRNAs on the prognosis of AML patients. It was discovered that the majority of circPLXNB2 was localized in the nucleus. CircPLXNB2 facilitated cell migration and proliferation while suppressing apoptosis, both *in vitro* and *in vivo*^39^. Additionally, knockdown of circSLC25A13 in AML cells reduced proliferation and elevated apoptosis both *in vitro* and *in vivo*^40^. Subsequent *in vitro* and *in vivo* research showed that knockdown of circ_0009910, which sponged miR-20a-5p, caused apoptosis and reduced AML cell proliferation^41^. Moreover, in AML cell lines and primary cells, circRNF220 selectively knockdown reduced proliferation and increased apoptosis^42^.

#### CircRNAs and AML cells cycle

In AML bone marrow samples and cells, the expression of circ_DLEU2 and cyclooxygenase 2 (COX2) was considerably higher than in controls. Knockdown of circ_DLEU2 in AML cells induced cell cycle arrest at the G0/G1 phase and promoted apoptosis, while also inhibiting cell proliferation^43^. It was discovered that circRNF220 was overexpressed in AML and targeted miR-330-5p to upregulate the expression of sex-determining region Y-related high-mobility group box 4 (SOX4). Silencing circRNF220 increased apoptosis while inhibiting AML cell proliferation, invasion, cell cycle progression, and glycolytic metabolism^44^. AML cell proliferation significantly decreased, the cell cycle was blocked to the G1 phase, apoptosis dramatically increased, and the capacity of AML cells to migrate and invade was significantly reduced when circRNF13 expression was downregulated^45^. Samples from patients with primary AML showed a consistent and substantial increase in SH3BGRL3 and circSH3BGRL3 expression as compared to healthy controls (HCs). CircSH3BGRL3 overexpression accelerated the cell cycle and promoted AML cell proliferation. The fraction of S phase cells increased, while the number of G0/G1 phase cells was considerably reduced with upregulation of circSH3BGRL3^46^.

#### CircRNAS and chemotherapy resistance

CircNPM1 was found to be abundantly expressed in serum samples from AML patients and AML cell lines, whereas MiR-345-5p, a target of circNPM1, was down-regulated in serum and cells. Silencing circNPM1 or restoring miR-345-5p inhibited colony formation, cell migration, and invasion; promoted apoptosis and cell-cycle arrest; and impaired Adriamycin (ADM) resistance in AML cells^47^. A doxorubicin-resistant THP-1 AML cell line (THP-1/ADM) was created by researchers in one study. The sensitivity of THP-1/ADM cells to ADM was considerably restored by down-regulating circPAN3 by small interfering RNAs, according to subsequent validation of the potentially functional circRNAs discovered in bone marrow tissues from 42 AML patients and in THP-1/ADM cells. Furthermore, a higher expression of circPAN3 was observed in bone marrow samples from individuals with refractory and relapsed AML^48^. Another experiment demonstrated that AML cells treated with Daunorubicin (DAU) exhibited a more pronounced proliferation arrest after knockdown of circSH3BGRL3 compared to the knockdown of circSH3BGRL3 alone. Silencing circSH3BGRL3 inhibited the activity of AML cells and enhanced their sensitivity to DAU^46^.

#### CircRNAS and the prognosis of AML patients

The study found that circ_001264 was abnormally overexpressed in AML patients and correlated with their poor prognosis^26^. CircSLC25A13 expression was elevated, supporting AML cell survival. Patients with poor overall survival (OS) had higher levels of circSLC25A13 (hsa_circ_0081188) compared to those with a better prognosis^40^. Comparing AML patients to those with iron deficiency anemia revealed that circ_0009910 was considerably elevated in the former group. High circ_0009910 expression in AML patients predicted a poorer risk and prognosis^41^. CircRNF220, with high sensitivity and specificity, can distinguish AML from acute lymphoblastic leukemia (ALL) and other hematological malignancies. It was shown to be more prevalent in the peripheral blood and bone marrow of juvenile AML patients. Interestingly, circRNF220 expression was not only a poor prognostic indicator for recurrence but also independently predicted overall prognosis^42^. Moreover, clinical outcomes for AML patients were shown to be negatively correlated with elevated circPLXNB2 levels^39^.

### CircRNAS were lowly expressed in AML

A new circRNA, circ_0001187, was found to be down-regulated in AML patients, and its low levels were associated with a poor prognosis. Additionally, it was discovered that, compared to controls, the expression of circ 0001187 was considerably higher in patients with hematological complete remission (HCR) and much lower in those with newly diagnosed (ND) AML. Knockdown of circ_0001187 markedly increased AML cell proliferation and prevented apoptosis both *in vitro* and *in vivo* through the miR-499a-5p/RNF113A/METTL3 pathway^49^. Analysis of AML cell lines and tissues revealed minimal expression of circCRKL. Additionally, the miR-196a-5p/miR-196b-5p/p27 axis demonstrated that circCRKL overexpression hindered AML cell proliferation and colony formation, whereas its silencing enhanced these functions^50^. Leukemia cells and AML patients both exhibited markedly reduced levels of circ_0040823. By targeting the miR-516b/PTEN axis, circ_0040823 overexpression induced apoptosis and cell cycle arrest while suppressing the growth of AML cells^51^. Additionally, the up-regulated hsa_circ_0121582 inhibited the proliferation of leukemia cells *in vitro* and *in vivo*^52^.

#### CircRNAs have potential associations with certain genes

Circ_0000370 was shown to be dramatically down-regulated in cells following the application of FLT3-ITD inhibitors and significantly up-regulated in FLT3-ITD+ AML patients 53. CircMYBL2 expression was shown to be higher in AML patients with FLT3-ITD+ than in those without FLT3-ITD+. Particularly, FLT3-ITD+ AML cell proliferation was suppressed and differentiation was enhanced both *in vivo* and *in vitro* by circMYBL2 knockdown. The researchers discovered an intriguing finding: CircMYBL2 significantly changed the mutant FLT3 kinase protein levels, which in turn activated the FLT3-ITD-dependent signaling pathway. Furthermore, inhibitor-resistant FLT3-ITD+ cells showed decreased cellular activity with circMYBL2 knockdown, accompanied by a notable decrease in FLT3 kinase expression and consequent deactivation of its downstream pathway^54^. In MLL-AF4 leukemia, a circRNA known as circAF4, derived from the AF4 gene, was shown to be a chaperone of the MLL fusion gene. Through the miR-128-3p/MLL-AF4 axis, circAF4 exhibited oncogenic functions in MLL-AF4 leukemia and stimulated leukemogenesis both *in vivo* and *in vitro*. Most notably, leukemic cells with the MLL-AF4 translocation showed an increase in apoptosis when circAF4 was knocked down, while leukemic cells without this translocation exhibited no change in apoptosis^55^. The circRNA circBCL11B, originating from the T-cell transcription factor gene B-cell CLL/lymphoma 11B, was shown to be expressed only in AML patients and not in healthy hematopoietic stem/progenitor cells (HSPCs). It was associated with a T-cell-like gene expression signature. Additionally, the researchers confirmed this finding in a separate sample of 332 AML patients. Furthermore, circBCL11B knockdown increased leukemia cell mortality and negatively impacted leukemia cell growth^56^.

Currently, little is known about the roles of circRNAs in the development and treatment of acute promyelocytic leukemia (APL). Detailed analysis showed that circRNAs expressed in APL cells were mostly exon-derived, not mere by-products of splicing, and could be distinguished from hematopoietic stem cells, neutrophils and lymphocytes. One of the circRNAs with variable expression, circ-HIPK2, had a major impact on the differentiation of APL cells induced by ATRA. Circ-HIPK2 may serve as a potential APL biomarker, as its expression in APL patients was notably lower than in normal peripheral mononuclear cells and other AML subtypes^57^.

AML is considered exceedingly complex in terms of disease biology, exhibiting substantial genetic, epigenetic, and phenotypic heterogeneity. During AML progression, molecular and cytogenetic alterations involve mutations in gene subpopulations that regulate normal cell proliferation, maturation, and survival^58^. Risk stratification for AML is not well defined, so further research into circRNAs is still needed to better integrate stratification with therapy guidance. The treatment of AML has also changed over the past decades, shifting from traditional cytotoxic chemotherapy to emerging targeted small molecule inhibitors, monoclonal antibodies, and epigenetic drugs, among others, which have drastically reduced treatment toxicity. Developing new drugs based on circRNAs is an avenue that deserves our continued efforts.

## CircRNAS and chronic myeloid leukemia

The fusion gene breakpoint cluster region - Abelson (BCR-ABL1) causes the myeloproliferative disorder known as chronic myeloid leukemia (CML)^59^. The unchecked tyrosine kinase activity of BCR-ABL1 contributes to the immortality of leukemia cells^60^. Imatinib (IM), which targets the BCR-ABL1 tyrosine kinase, is currently one of the first-line choices in the treatment of CML^61^. However, treatment resistance remains a persistent issue in the pursuit of a cure. The following section summarizes the impact of circRNAs on drug resistance in the CML treatment in recent years. The possibility of producing new circRNAs through fusion is clear. Upon review, it was found that most circRNAs typically existed in the coding region of genes^62^.

### Two specific sources of circRNA

CircBA9.3, a unique circular RNA derived from BCR-ABL1, which had the ability to both efficiently prevent and enhance cell apoptosis. A favorable correlation was observed between the BCR-ABL1 levels and increased circBA9.3 expression in some individuals resistant to tyrosine kinase inhibitors (TKIs). Furthermore, circBA9.3 can increase the expression of the oncoproteins c-ABL1 and BCR-ABL1, with its primary location being in the cytoplasm. Tyrosine kinase activity was elevated in circBA9.3, a factor that may contribute to resistance to TKI treatment^60^. Another novel fused circRNA, F-circBA1, derived from BCR-ABL in K562 and K562/G01 cells, was also primarily localized in the cytoplasm. Knockdown of F-circBA1 by shRNA inhibited the proliferation of K562 and K562/G01 cells, resulting in cell cycle arrest at G2/M phase. In addition, results from the murine leukemogenesis model showed that F-circBA1 knockdown inhibited leukemogenesis *in vivo*^63^.

### Most circRNAs are not generated by classical translocation^59^

According to research, the downregulation of the mammalian sirtuin 1 (SIRT1) protein during senescence and *in vivo* ageing was mediated by macroautophagy (henceforth referred to as autophagy), a catabolic membrane trafficking mechanism that broke down cellular components through autophagosomes and lysosomes^64^. SIRT1 gave rise to a new circRNA called circ_SIRT1, which was highly expressed in CML and linked to medication resistance. The viability, invasion, and apoptosis of K562/R cells were modulated by circ_SIRT1 knockdown. Additionally, the inhibition of circ_SIRT1 decreased the IC50 of K562/R cells to IM and reduced the degree of autophagy. The mechanism by which circ_SIRT1 affected autophagy level and IM resistance was by its direct binding to the transcription factor Eukaryotic Translation Initiation Factor 4A3 (EIF4A3), which in turn regulated the transcription of Autophagy Related 12 (ATG12) mediated by EIF4A3^65^. Moreover, autophagy activation coincided with the development of CML treatment resistance. Patients with CML who were resistant to IM had elevated levels of circ 0009910 in their blood and K562/R cells. In K562/R cells, knockdown of circ_0009910 led to increased apoptosis and inhibition of both autophagy and proliferation. It has been demonstrated that circ 0009910 increased IM-resistance in CML cells via modifying ULK1-induced autophagy through targeting miR-34a-5p^66^. In BCR-ABL + cells, overexpression of circCRKL, which was generated from the CML-related gene CRKL. Knockdown of circCRKL increased the sensitivity of resistant cells to IM and reduced BCR-ABL + cell growth both *in vitro* and *in vivo*^67^.

### One circRNA may act on multiple signaling pathways

Based on their sensitivity to IM, the study recruited 108 CML patients and divided them into two groups: a responsive group (n = 66) and a non-responsive group (n = 42). The analysis revealed that the non-responsive group had considerably lower expression of miR-203 and miR-637, and significantly higher expression of circ_0080145, circ_0051886, and ABL proto-oncogene1 (ABL1) mRNA compared to the responsive group. Meanwhile, ABL1 was discovered to be the common direct target gene of miR-203 and miR-637, confirming predictions that circ_0080145 and circ_0051886 targeted miR-203 and miR-637, respectively. The dysregulation of these two circRNAs led to the development of IM resistance in CML, at least partially^61^. Previous *in vitro* experiments have demonstrated that circ_0080145 promoted IM resistance by regulating the miR-326/PPFIA1 axis. Patients and cells with IM-resistant CML have elevated levels of circ_0080145. *In vivo*, silencing of circ_0080145 resulted in death in IM-resistant CML cells and decreased cell proliferation, glycolysis, and IM resistance. Furthermore, circ_0080145 knockdown prevented IM resistance and inhibited tumor development *in vivo*^68^. Previous research investigated the circRNA expression profile in CML patients and established a thorough regulatory network for the competitive endogenous RNA (ceRNA) controlled by hsa_circ_0080145. Additionally, functional tests showed that knockdown of hsa_circ_0080145 significantly inhibited CML cell growth by acting as a miR-29b sponge^69^.

### CircRNAs were upregulated in peripheral blood monocytes and/or serum

In a study involving 100 CML patients and age-matched healthy donors (HDs), circHIPK3 was upregulated in CML patients (p<0.001). Serum circHIPK3 levels were independently predictive of prognosis in CML patients (p=0.02) and demonstrated a strong connection (p=0.01) with patient prognosis. Moreover, patients with high circHIPK3 expression had shorter OS rates (p=0.002). Based on the above evidence, elevated serum circHIPK3 levels were associated with adverse outcomes in CML patients. Knockdown of circHIPK3 significantly inhibited CML cell proliferation and induced apoptosis^70^. Additionally, it was discovered that peripheral blood mononuclear cells (PBMCs) and CML serum samples had much higher levels of circ_100053 compared to HCs. The high expression of circ_100053 was associated with a poor prognosis and increased resistance to imatinib (IM) in CML patients^71^. Conversely, low the clinical effectiveness of imatinib was linked to an increase in hsa_circ_0058493 expression specifically in peripheral blood monocytes. Additionally, the suppression of circ_0058493 expression dramatically slowed the growth of IM-resistant CML cells^72^.

Currently, clinical treatment with combination TKIs is highly effective in most CML patients, but it does not cure the disease, and some patients experience intolerance or resistance. The resistance or intolerance to these inhibitors is what ultimately drives disease progression^73^. Therefore, as our understanding of circRNAs continues to grow, ongoing efforts are essential to explore new potential therapeutic strategies.

## CircRNAs in lymphoid malignancies

Lymphoid malignancies, a category of clonal cancers, originate at various stages of B and T cell development. This article focuses on the role of circular RNAs (circRNAs) in the progression of acute lymphoblastic leukemia (ALL), chronic lymphocytic leukemia (CLL), lymphoma, and multiple myeloma (MM).

### CircRNAs and ALL

ALL is an aggressive neoplasm that includes both B-cell acute lymphoblastic leukemia (B-ALL) and T-cell acute lymphoblastic leukemia (T-ALL)^74^. Nonetheless, T-ALL remains a primary focus of contemporary circRNAs research. It was observed that circ_0008012 was upregulated in MLL/AF4 + ALL cells, and knocking down circ_0008012 inhibited proliferation and promoted the apoptosis in these cells^75^. Circ_0000094 was primarily located in the cytoplasm, and its expression was significantly reduced in T-ALL. Overexpression of circ_0000094, through regulation of the miR-223-3p/FBW7 axis, markedly inhibited T-ALL cell proliferation, migration, and invasion, while promoting cell death^76^. Circ 0000745 and notch receptor 1 (NOTCH1) were shown to be overexpressed in both T-ALL bone marrow and T-ALL cells, contributing to tumor development. Acting as a miR-193b-3p sponge in T-ALL, circ 0000745 upregulated NOTCH1, thereby promoting cell proliferation and inhibiting apoptosis^77^. SOX4 and circPRKCI were both overexpressed in clinical T-ALL samples. Although miR-20a-5p expression was inversely related to the expression of both SOX4 and circPRKCI, the expression of SOX4 and circPRKCI was positively correlated. Meanwhile, silencing circPRKCI increased the expression of miR-20a-5p in T-ALL cells, which in turn accelerated apoptosis in these cells^78^. Circ0000745 was shown to be elevated in ALL cell lines and patients, contributing to the progression of the disease. Knockdown of circ_0000745, by modulating the miR-494-3p/NET1 axis, induced cell death and ferroptosis, reduced glycolysis, and inhibited cell cycle progression^79^. Increased expression levels of circ-PRKDC and Reelin (RELN) and decreased expression levels of miR-653-5p were measured in T-ALL tissues and cells from patients (n=39) compared to HCs (n=30). Cellular experiments revealed that silencing of circ-PRKDC or enforcing the expression of miR-653-5p inhibited RELN expression and activation of the PI3K/AKT/mTOR signaling pathway, which in turn enhanced cell autophagy and apoptosis while disrupting cell proliferation^80^. CircADD2 was demonstrated to be downregulated in ALL tissues and cell lines. However, when overexpressed *in vitro* and *in vivo*, circADD2 acted as an oncogene by sponging miR-149-5p, which promoted apoptosis and inhibited cell growth^81^. The increased expression of circ-0000745 in leukemia cells (Kasumi-1 and KG-1) led to increased activation of the extracellular signal-regulated kinase (ERK) pathway. This activation resulted in a decrease in apoptosis and a significant increase in the proliferation of Kasumi-1 and KG-1 cells^82^. Experiments demonstrated circPVT1 enhanced the activation of the NF-κB signaling pathway by downregulating miR-125b, which in turn promoted the proliferation, migration, and invasion of ALL cells^83^. Interestingly, T-ALL bone marrow and cell lines exhibited markedly elevated circPVT1 expression levels. In T-ALL cell lines, knockdown of circPVT1 enhanced apoptosis and reduced cell proliferation via the miR-30e/DLL4 pathway. Moreover, the 5-year survival rate and cumulative relapse rate were strongly correlated with circPVT1 levels^84^. In a different investigation, ALL bone marrow samples showed higher levels of circPVT1 expression compared to normal bone marrow samples. Similarly, a significant increase in circPVT1 expression was observed in ALL cell lines.

Knockdown of circPVT1 suppressed its neighboring gene, cellular Myelocytomatosis oncogene (c-Myc), and reduced the production of the antiapoptotic Bcl-2 protein, leading to decreased cell proliferation and induced apoptosis^85^. The expression of circFBXW7 varies significantly among T-ALL patients. Reduction of circFBXW7 was shown to enhance the viability and proliferation of leukemia cells. Additionally, it was discovered that T-ALL cells became more sensitive to dexamethasone when circFBXW7 levels was reduced. Moreover, the proliferative phenotype linked to the abnormal depletion of circFBXW7 in T-ALL contributed to the hyperactivation of MYC and NOTCH1, both of which are crucial for the etiology and progression of leukemia^86^. Myelosuppression is well known to be a common side effect of chemotherapy. In a study involving 60 patients with ALL who received platelet transfusions, researchers identified 257 circRNAs potentially associated with platelet transfusions in ALL by constructing circRNA expression profiles and screening for differentially expressed circRNAs^87^.

The review found that the research on circRNAs has been primarily focused on T-ALL, with limited investigations in B-ALL, Philadelphia chromosome-like acute lymphoblastic leukemia (Ph-like ALL), and Philadelphia chromosome-positive acute lymphoblastic leukemia (Ph+ ALL). Additionally, further research is needed to explore the connection between circRNAs and the anemia and leukopenia observed in myelosuppression following chemotherapy, as well as thrombocytopenia.

### CircRNAs and chronic lymphocytic leukemia

Chronic lymphocytic leukemia (CLL) is a blood disorder characterized by the clonal proliferation of mature B lymphocytes in the peripheral blood, bone marrow, spleen, and lymph nodes, typically occurring in middle-aged and older adults^88^. The landscape of CLL therapy has radically changed with the advent of next-generation anti-CD20 monoclonal antibodies and highly effective oral targeted treatments^89^. Therefore, could the current studies on circRNAs in CLL point us in a new direction??

Research indicated that circ-RPL15 was considerably more abundant in CLL plasma and PBMC samples compared to normal control samples. With an area under the ROC curve (AUC) of 0.84, circ-RPL15 emerged as a promising biomarker for CLL screening. Furthermore, OS was significantly lower for CLL patients with high circ-RPL15 expression compared to those with low circ-RPL15 expression. It was also discovered that when circ-RPL15 expression was suppressed, miR-146b-3p inhibited the RAS/RAF1/MEK/ERK pathway, leading to decreased CLL cell survival^90^. Data showed that OS was significantly shorter in patients with increased circTET2 expression. Moreover, a higher percentage of patients in the high circTET2 group experienced relapse or therapeutic resistance. CircTET2 overexpression activated the mechanistic target of rapamycin complex 1 (mTORC1) pathway and elevated the levels of carnitine palmitoyl transferase 1A (CPT1A) and carnitine palmitoyl transferase 1B (CPT1B), which in turn promoted the growth of CLL cells^91^. This study demonstrated a correlation between shorter OS and increased expression of circ_0002078 in ALL patients. Aberrantly high expression of circ_0002078 has been shown to reduce miR-185-3p binding to transcription factor 7-like 1 (TCF7L1), leading to the up-regulation of TCF7L1. This up-regulation promoted cell proliferation, metastasis, and other biological processes, thereby contributing to the progression of CLL^92^. A strong correlation was found between circRIC8B expression levels and elevated low-density lipoprotein cholesterol (LDL-C) in individuals with CLL. Patients with increased circRIC8B expression had a worse prognosis and shorter survival, according to research on PBMC samples from 63 CLL patients. Through the miR-199b-5p/LPL axis, knockdown of circRIC8B significantly reduced CLL cell proliferation and lipid buildup. CircRIC8B acted as a sponge for miR-199b-5p, preventing it from reducing lipoprotein lipase (LPL) mRNA levels. Furthermore, ezetimibe inhibited the development of CLL cells and reduced LPL mRNA expression^93^. Due to its substantial overexpression in CLL cells, circ-CBFB may serve as a biomarker for both prognosis and diagnosis in CLL patients. By blocking the miR-607/FZD3/Wnt/β-catenin pathway, circ-CBFB expression dramatically reduced CLL cell proliferation, inhibited cell cycle progression, and triggered apoptosis^94^. Furthermore, circ 0132266 was found to regulate the miR-337-3p/PML axis, stimulating CLL cell death and inhibiting cell proliferation 95. In CLL samples, circZNF91 expression was elevated. Silencing circZNF91 through the miR-1283/WEE1 axis can reduce the proliferation of CLL cells, induce apoptosis, and cause cell cycle arrest^96^. The biological and clinical characterization of mitochondrial genome-derived (mt)-circRNAs in CLL remain largely unknown. However, it was found that mc-COX2 (mitochondrial genome-derived circRNAs [mc]) was abundant in exosomes and can be delivered from CLL cells to plasma. Mc-COX2 not only regulated mitochondrial function, but a decrease in its levels also markedly induced apoptosis. Furthermore, mitochondria-related compounds and their inhibitors reduced mc-COX2 levels and inhibited CLL cell proliferation. In addition, CLL patients with TP53 deletions, but not those with TP53 mutations, showed higher mc-COX2 expression^97^.

Numerous therapeutic options are available for the treatment of CLL; nevertheless, drug resistance remains a significant challenge. Further research is needed to determine the long-term prognosis and uncover potential harmful effects of multidrug combinations. Furthermore, mutations in the TP53 gene and/or deletion of the short arm of chromosome 17 (del[17p]) are associated with resistance to chemotherapy and a shortened time to progression on the majority of targeted treatments^98^. Consequently, future research on circRNAs in CLL may focus on prognosis, staging, and drug resistance.

### CircRNAs and lymphoma

Lymphomas, a diverse category of lymphoid cancers, exhibit a wide range of clinical behavior patterns and variations in treatment response. Prognosis is influenced by histologic type, clinical variables, and, more recently, molecular features^99^. In human diffuse large B-cell lymphoma (DLBCL) specimens and cultured DLBCL cells, circPCBP2 was elevated, while miR-33a/b was downregulated. Patients with high circPCBP2 levels had significantly poorer survival rates compared to those with low circPCBP2. CircPCBP2 may negatively regulate miR-133a/b expression, and miR-133a/b directly targeted PD-L1 mRNA to inhibit PD-L1 signaling. By sponging miR-33a/b and thereby disinhibiting PD-L1 expression, circPBCP2 knockdown enhanced the anti-tumor effects of cyclophosphamide, doxorubicin, vincristine, and prednisone (CHOP) and inhibited DLBCL development *in vivo*^100^. The expression of circ_0003645 was markedly elevated in the tumor tissues of DLBCL. Knockdown of circ_0003645 inhibited DLBCL cell viability, disrupted cell cycle and glycolysis, and promoted apoptosis through the miR-335-5p/NFIB axis^101^. It was discovered that DLBCL expressed circ_OTUD7A at high levels, leading to the upregulation of forkhead box protein 1 (FOXP1). By controlling the miR-431-5p/FOXP1 axis, knockdown of circ_OTUD7A facilitated cell cycle arrest and induced cell death while suppressing cell proliferation and metastasis^102^. In DLBCL, circSPEF2 expression was abnormally downregulated. By targeting the miR-16-5p/BACH2 axis, increasing circSPEF2 expression reduced the number of Treg cells and increased the proportion of CD4T cells in the tumor microenvironment.

This shift suppressed the proliferative activity and induced apoptosis of lymphoma cells both *in vitro* and *in vivo*^103^. It was discovered that DLBCL tissues expressed higher levels of circZNF609 compared to controls. Downregulation of circZNF609, targeting miR-153, induced apoptosis and inhibited the growth of DLBCL OCI-LY19 cells.^104^. Circ-APC (hsa_circ_0127621) was downregulated in DLBCL tissues, cell lines, and plasma. It was shown that circ-APC served as a miR-888 sponge, and ectopic expression of circ-APC slowed the development of tumors *in vivo* and inhibited the proliferation of DLBCL cells *in vitro*^105^. Additionally, circCFL1 overexpression promoted the proliferation and migration of DLBCL cells by regulating high mobility group box 1 (HMGB1) expression through miR-107^106^.

The development of therapeutic methods for DLBCL is an ongoing process. Currently, circRNA research in DLBCL primarily focuses on cellular studies, and it is anticipated that this research will lead to novel treatment options.

#### Several specific types of lymphomas

##### Epstein-Barr virus positive diffuse large B-cell lymphoma

CircRNAs have been studied in Epstein-Barr virus positive DLBCL (EBV + DLBCL) to explore their impact on the sensitivity of these cells to chemotherapeutic agents. Researchers discovered that in EBV + DLBCL, circEAF2 was downregulated and negatively correlated with both the development of DLBCL and EBV infection. Overexpression of circEAF2, through the miR-BART19-3p/APC/β-catenin axis, induced apoptosis and increased the sensitivity of lymphoma cells to epirubicin^107^.

##### Mantle cell lymphoma

Poor patient survival in mantle cell lymphoma (MCL) was predicted by the upregulation of circCTNNA1, which may also act as a sponge for miR-34a, thereby promoting the growth of cancer cells. Poor survival was indicated by high circCTNNA1 expression levels in MCL patients^108^. Compared to HDs, the plasma of MCL patients exhibited higher levels of circCDYL expression. The ROC curve analysis indicated that circCDYL had significant diagnostic value, with an AUC of 0.856. Additionally, experimental evidence demonstrated that circCDYL knockdown inhibited the growth of MCL cells^109^.

##### Natural killer/T-cell lymphoma

The relative expression of circADARB1 was elevated in the plasma of patients with Natural killer/T-cell lymphoma (NKTCL), suggesting its potential utility in aiding the diagnosis and prediction of NKTCL responses. Knockdown of circADARB1 was found to inhibit NKTCL cell proliferation both *in vitro* and *in vivo*^110^. In NKTL cell lines, circKIF4A was found to be markedly elevated, and this overexpression was associated with a poor prognosis for NKTL. NKTL cells' glucose uptake and lactate production were markedly reduced by circKIF4A silencing via sponge miR-1231 modulation of Pyruvate dehydrogenase kinase 1 (PDK1) and B-cell lymphoma/leukemia 11A (BCL11A) levels^111^.

##### T-cell lymphoblastic lymphoma

Research revealed that various circRNAs were differentially expressed in T-cell lymphoblastic lymphoma (T-LBL) tissues compared to normal thymic tissues, with Circ-LAMP1 being the most highly expressed circRNA in the cancerous tissues. Meanwhile, the upregulation of circ-LAMP1 in T-LBL tissues and cell lines was further verified. It has been established that circ-LAMP1 stimulated cell proliferation via the miR-615-5p/DDR2 signaling pathway^112^.

In summary, much remains to be learned about circRNAs in rare types of lymphomas, as they have not yet been thoroughly investigated.

## CircRNAs and multiple myeloma

As the second most common hematological malignancy, multiple myeloma (MM) is characterized by unchecked plasma cell proliferation, typically leading to elevated calcium levels, renal insufficiency, anemia, bone disease^113^, ^114^. Despite notable progress over the last two decades, MM remains an incurable disease^115^. Thanks to recent advances in our understanding of circRNAs, they now significantly influence the diagnosis, prognosis, and clinicopathologic features of MM^116^.

### CircRNAs may have a role in the MM pathogenesis

Both the bone marrow cells and the plasma showed a substantial rise in circ-CDYL levels. As a result of circ-CDYL's ability to absorb miR-1180 and lessen its suppression of yes-associated protein (YAP), YAP expression was elevated, which in turn caused uncontrollable proliferation of MM cells^117^. Compared with normal samples, circ-MYBL2 was significantly reduced in MM tissue and serum samples, and low circ-MYBL2 level was strongly linked to unfavorable outcomes and advanced clinical staging. Subsequent investigations revealed that circ-MYBL2 suppressed tumor growth by modifying the phosphorylation levels of the linear MYBL2 isoform.

By promoting the binding of cyclin F to bind to V-Myb avian myeloblastosis viral oncogene homolog-like 2 (MYBL2), circ-MYBL2 dampened the phosphorylation and activation of MYBL2, which in turn prevented the transcription of several well-known oncogenes associated with proliferation^118^. Compared to normal plasma cell lines and healthy volunteers, MM patients' BM-derived plasma cells and MM cells lines expressed higher levels of circ_0007841. circ_0007841 acted as an oncogene by sequestering miR-338-3p to upregulate the expression of Bromodomain-containing protein 4 (BRD4), thereby promoting the proliferation, cell cycle progression, and motility of MM cells while inhibiting their apoptosis^119^. The findings demonstrated that circXPO1 was elevated in MM cell lines and tissues. Silencing circXPO1 inhibited cell proliferation and promoted apoptosis, resulting in G1 phase arrest through the miR-495-3p/DDIT4 axis^120^.

### CircRNAs may contribute to poorer prognosis in MM patients

Results from a study including 136 MM patients and 74 HCs revealed that hsa_circ_0087776 was consistently expressed in MM cells but was significantly lower in the serum of MM patients. Furthermore, the joint diagnosis using albumin (ALB) and beta2-Microglobulin (β₂-MG) was significantly enhanced, providing a foundation for diagnosis^121^. The study found the top 10 upregulated and downregulated circRNAs associated with tumor pathways, including the vascular endothelial growth factor (VEGF) and mitogen-activated protein kinases (MAPK) signaling pathways, in 60 MM patients and 30 HCs. Further validation revealed that circ-PTK2 and circ-RNF217 were linked to poor treatment response and survival, whereas circ-AFF2 predicted favorable treatment response and survival in MM patients^122^. It was found that circ_0000190 levels were reduced in MM patients compared to HDs. Regarding clinical response, circ_0000190 showed predictive value for an increased overall response rate (ORR), and its high expression of circ_0000190 was associated with better progression-free-survival (PFS) and OS. Moreover, high expression of circ_0000190 was identified as an independent factor predicting better OS^123^. Among 60 MM patients who received induction therapy with bortezomib (BTZ), 8 patients with complete remission (CR) and 8 patients with no response (NR) were randomly selected. The study identified circ_0026652 as a critical prognostic factor, concurrently linked to CR, ORR, PFS, and OS. In U266 and RPIM-8226 cells, knockdown of circ_0026652 was shown to increase sensitivity to BTZ through the Wnt/β-catenin pathway, regulated by microRNA (miR)-608^124^. The expression of circ_0001821 was elevated in MM cell lines and bone marrow tissues compared to normal controls (NCs). MM cells with overexpression of circ_0001821 exhibited enhanced proliferation and delayed apoptosis. In patients aged 60 years or older, high circ 0001821 expression was associated with worse OS in contrast to low circ 0001821 expression. Moreover, elevated circ_0001821 expression served as an independent prognostic factor for the unfavorable OS^125^. When comparing circulating exosomes from MM patients who were resistant to BTZ, the expression of circMYC was found to be significantly higher. Moreover, the expression level of exosomal circMYC was associated with the International Staging System, t(4;14) translocation, Durie-Salmon staging, and deletion 17p. Patients with elevated exosomal circMYC expression experienced higher relapse and mortality rates, and this marker independently predicted a poor prognosis for MM patients^126^. The expression level of circ_0007841 in MM patients was found to be higher than in HCs but decreased following bortezomib (BTZ)-based induction treatment. Furthermore, circ 0007841 expression at diagnosis was associated with t (4:14) and was an independent predictor of shorter PFS and OS in MM patients^127^. Interestingly, another study found that upregulation of hsa_circ_0007841 was associated with a poorer prognosis in MM patients^128^. In MM patients, circ_0000142 was highly expressed, and this elevated expression was strongly correlated with advanced Durie-Salmon stage and higher International Staging System scores^129^. An increase in circBUB1B_544aa in peripheral blood samples was strongly associated with poor outcomes in MM. Additionally, circBUB1B_544aa, in concert with BUB1 mitotic checkpoint serine/threonine kinase B (BUB1B), contributed to the induction of chromosomal instability (CIN). Interestingly, MM malignancy was markedly reduced both *in vitro* and *in vivo* by BUB1B siRNA, which targeted the kinase catalytic site of both BUB1B and circBUB1B_544aa^130^. Samples from multiple myeloma (MM) patients exhibited significantly higher levels of Checkpoint Kinase 1 (CHEK1) expression than normal plasma cells, and elevated CHEK1 expression were linked to worse outcomes in MM patients. Furthermore, increased CHEK1 expression promoted MM cell growth and treatment resistance both *in vitro* and *in vivo*. Similarly, the overexpression of circCHEK1_246aa, a circular RNA encoding the catalytic core of CHEK1 kinase, enhanced MM cell invasiveness and promoted osteoclast growth^131^. The expression of circ-ATP10A in the peripheral blood of MM patients was significantly increased. This circRNA can act as a microRNA sponge, regulating downstream mRNA (such as VEGFB, vascular endothelial growth factor B; HIF1A, hypoxia inducible factor 1 subunit alpha; PDGF, Platelet-derived growth factor and FGF, fibroblast growth factor), thereby promoting angiogenesis in MM^132^. Immunoglobulin D type MM (IgD MM) is a relatively uncommon yet highly severe subtype of all MM cases. In contrast to lgG and normal plasma cell samples (NPCs), circHNRNPU was one of the most prevalent and differently expressed circRNAs in IgD MM, and its increased expression was linked to unfavorable outcomes^133^.

One terrible side effect of MM is myocardial damage, an irreversible condition that impairs quality of life and patient outcomes. Research has found that the level of circ-CACNG2 in the serum exosomes of MM patients was relatively high. Circ-CACNG2 was identified as an independent predictive and diagnostic indication for cardiac problems connected to MM through clinical data analysis. Furthermore, *in vitro* studies shown that MM-exosomes might partly activate the circ-CACNG2/miR-197-3p/caspase3 axis, leading to reduced cardiomyocyte survival and increased apoptosis^134^. In a trial including 6 MM patients and 5 HCs, circ-G042080 was observed to be substantially expressed in the serum exosomes of MM patients. Circ-G042080 was shown to potentially cause MM-related myocardial injury through the downstream miRNA/TLR4 axis. Additionally, the expression level of circ-G042080 was strongly correlated with the clinical severity of MM and the extent of MM-related myocardial damage^135^.

### CircRNAs were associated with therapeutic drug resistance

As a miR-331-3p sponge, circPSAP was overexpressed in MM patients, and its elevated levels were associated with a poor prognosis. In the meantime, the suppression of histone deacetylase 4 (HDAC4) by miR-331-3p reduced cell growth, increased cell death and enhanced BTZ sensitivity^136^. Patients and cells with BTZ-resistant MM exhibited up-regulated circ-CCT3 and BRD4, and down-regulated miR-223-3p. These findings demonstrated that circ-CCT3 regulated the miR-223-3p/BRD4 pathway, which in turn influenced BTZ resistance in MM^137^. CircRERE was upregulated in BTZ resistant MM samples and cells and inhibited BTZ resistance in MM cells by mediating the miR-152-3p/CD47 axis^138^. Doxorubicin-resistant cells showed an upregulation of hsa_circ_0007841 compared to parent cells. In these resistant cells, there was a simultaneous increase in the messenger RNA and protein levels of ATP-binding cassette transporter G2 (ABCG2). More significantly, discrepancies in half-maximal inhibitory concentration between parent and drug-resistant cell lines may be reduced by inhibiting ABCG2^139^. A downregulated expression of circITCH in MM bone marrow tissues, cell lines, and BTZ-resistant MM cells indicated an adverse prognosis for MM patients. Experiments have demonstrated that circITCH overexpression increased the susceptibility of MM cells to BTZ via the miR-615-3p/PRKCD axis Both *in vitro* and *in vivo*^140^. MM tissues and cell lines exhibited an upregulation of circ_0005615, which promoted malignant development and BTZ resistance via facilitating miR-185-5p/IRF4 signaling^141^. A study observed the expression of hsa_circ_0007841 in the tissues of 86 MM patients. The expression of hsa_circ_0007841 was shown to be significantly increased in both MM and BTZ-resistant cell lines, a finding strongly associated with the illness's prognosis. In particular, chromosomal abnormalities including gain 1q21, t (4:14), and mutations in the ATR and IRF4 genes, were linked to hsa_circ_0007841 overexpression^128^.

### CircRNAs may affect a range of MM cells activities

Circ 0007841 was discovered in bone marrow aspirates and cells from MM patients. Knockdown of circ 0007841 inhibited the development, metastasis, and resistance to therapy of MM cells, while also promoting apoptosis of MM cells *in vitro* and decreasing tumor growth *in vivo* by modulating the miR-129-5p/JAG1 axis^142^. In bone marrow tissues and peripheral blood, circ_0000190 was downregulated and localized in the cytoplasm. Furthermore, by negatively regulation of miR-767-5p, circ_0000190 decreased cell viability, proliferation, while triggering apoptosis in MM cells, thereby reducing cell progression. The findings showed that circ_0000190 repressed miR-767-5p, conferring protection against MM. Additionally, miR-767-5p may function as an oncogenic driver by targeting mitogen-stimulated protein kinase 4 (MAPK4)^143^. Circ-PTK2 was overexpressed in most multiple myeloma (MM) cell lines compared to normal plasma cells. Circ-PTK2 activated WNT/β-catenin and MEK/ERK signaling pathways through miR-638, promoting cell migration and proliferation while inhibiting apoptosis^144^. Aspirates and cells from MM bone marrow showed an upregulation of circ_0058058. Silencing circ_0058058 decreased the development and proliferation of MM tumors, however, *in vitro* it induced apoptosis^145^. In MM tissues, circ_0003489 and Pre-B-cell leukemia homeobox 3 (PBX3) were upregulated, while miR-433-3p was downregulated. *In vitro*, overexpression of miR-433-3p or knockdown of circ_0003489 significantly reduced MM cell proliferation and boosted apoptosis^146^. It was discovered that circKCNQ5 competitively sponged miR-335-5p, thereby accelerating the progression of MM. *In vitro*, knockdown of circKCNQ5 increased MM cell apoptosis, while suppressing proliferation, migration, invasion, and glycolysis^147^. Research has demonstrated that circ_SEC61A1 levels were elevated in MM tissues and cells, predominantly localized in the cytoplasm. In MM cells, silencing circ_SEC61A1 accelerated apoptosis, reduced metastasis, and decreased proliferation^148^. The levels of circ_0005615 and IGF1R were elevated in MM patients and cells. By targeting the miR-331-3p/IGF1R axis, the downregulation of circ 0005615 accelerated MM cell death while delaying proliferation and cell cycle progression^149^. CircATIC partially sponged miR-324-5p, leading to increased expression of hepatocyte growth factor (HGF) in MM cells. MiR-324-5p decreased the malignant tendencies of MM cells; however, these effects were largely offset by the cells' overexpression of HGF. Thus, by regulating miR-324-5p/HGF signaling, circATIC promoted the proliferation, migration, invasion, and glycolysis in MM cells while suppressing apoptosis^150^. In MM cells *in vitro* and MM xenografts *in vivo*, suppression of hsa_circ_0003489 strongly reduced cell viability, proliferation, and autophagy via the miR-874-3p/HDAC1 axis, while promoting apoptosis. Additionally, inhibition of hsa_circ_0003489 enhanced the cytotoxic effects of BTZ in MM cells and reversed BTZ-induced autophagy^151^. The expression of circ_0111738 was low in MM patients and cells. Overexpression of circ_0111738 inhibited proliferation, migration, invasion, and angiogenesis in MM cells. Furthermore, *in vivo* anti-tumor effects of circ_0111738 overexpression were also observed^152^. Low levels of neuron-derived neurotrophic factor (NDNF) and circ_0134426 were observed in the nude mouse xenograft model, and similar findings were noted in MM bone marrow samples. By regulating the miR-146b-3p/NDNF network, overexpression of circ_0134426 hampered tumor establishment in xenograft models and inhibited the proliferation, colony formation, and migration of MM cells^153^. In a study of 66 MM patients and 21 normal controls, the expression of circ_0069767 was found to be significantly higher in MM patients compared to the normal controls. Cell function experiments demonstrated that circ_0069767 regulated the expression of K-RAS by sponging miR-636 in MM cells. Overexpression of circ_0069767 lead to reduced proliferation, migration, and invasion of MM cells, while increasing apoptosis, thereby exhibiting anti-tumor effects^154^.

Reviewing the research of circRNAs in MM in recent years found a wide range of aspects involved, but circRNAs are still in an unfamiliar field and still need our further efforts to explore. Research has primarily focused on examining the relationship between circRNAs and drug resistance to BTZ in MM. Consequently, further research is necessary to fully understand circRNAs and other drug resistance. Simultaneously, the staging of MM diagnosis is crucial, and investigations are still needed to determine if circRNAs may be utilized as a staging indicator. Additionally, whether circRNAs produce the same effects across different types of MM still needs further explored.

## Summary and prospects

After investigations, we have uncovered the deep and complex role that circRNAs play in the biology of hematological malignancies. These non-coding RNAs have become important regulators of gene expression, intriguing biomarkers, and potential targets for treatment because of their covalently closed loop topologies. In light of the roles that circRNAs play in hematological malignancies, this discussion aims to highlight key findings, provide insights, and offer recommendations for future research.

CircRNAs are important in hematological malignancies because of their complex involvement in controlling cellular processes, which are essential to the progression of cancer, including apoptosis, proliferation, and metastasis. A sophisticated layer of post-transcriptional control is shown by their capacity to function as miRNA sponges, influencing the translation and stability of mRNAs. This ability might be used for therapeutic purposes. The prognosis, treatment resistance, and etiology have all been linked to circRNAs. It appears possible to build circRNA-based diagnostics and prognostics because of the specificity of circRNAs expression patterns across a range of malignancies and stages.

The translation of circRNAs research into clinical applications is hampered by a number of issues, despite the encouraging evidence suggesting that *in vitro* and *in vivo* models of hematological malignancies will be crucial in verifying circRNAs activity and therapeutic potential. The biosynthesis, regulation, and function of circRNAs are still not fully known, which calls for more research. Second, the cellular environment and illness state, as well as the dynamic and context-dependent activities of circRNAs, make it more difficult to identify targets for treatment. Another major obstacle is the creation of effective and focused delivery mechanisms for circRNA-based treatments. Moreover, the feasibility of circRNA-based interventions as therapies will also be evaluated via clinical studies concentrating on them.

There will be plenty of opportunities in the field of circRNAs research in hematological malignancies in the future. Advanced sequencing and bioinformatic analysis will continue to reveal new circRNAs and their role in hematological malignancies. Functional studies provide light on the molecular mechanisms underlying circRNAs activity, including their interactions with proteins and miRNAs. Furthermore, the creation of circRNAs mimics or inhibitors in conjunction with tailored delivery methods may open up new therapeutic possibilities.

In conclusion, circRNAs have opened up an intriguing and promising new area for the research and treatment of hematological malignancies. Their work sheds light on the complexity of cancer biology and opens the way to novel therapeutic and diagnostic strategies. Overcoming the current barriers will need a focused effort from varied teams and may result in improved patient outcomes and new paradigms in cancer therapy. As our knowledge of cancer advances, circRNAs should play a bigger role in the battle against the hematological malignancies.

## Figures and Tables

**Table 1:** Overview of the potential effects of circRANS in AML and APL.

CircRNA	Expression	Function	miRNAs, potential mechanism	Reference
Circ_0009581	↑	tumor development (+)	miR-150-5	25
Circ_0001947	↓	tumor development (+)	miR-454-3p	25
Circ_001264	↑	tumor load decreased	regulated the RAF1 expression and activated the p38-STAT3 signaling pathway	26
CircZBTB46	↑	knockdown: cell cycle arrest (+), ferroptosis (+), proliferation (-)	upregulated the expression of stearoyl-CoA desaturase 1 (SCD)	27
CircSLC25A13	↑	cell proliferation (+), apoptosis (+)	miR-616-3p	40
Circ_0009910	↑	knockdown: cell apoptosis (+), proliferation (-)	miR-20a-5p	41
CircRNF220	↑	knockdown: cell apoptosis (+), proliferation (-)	miR-30a	42
CircPLXNB2	↑	knockdown: cell apoptosis (+), cycle arrest (+), proliferation (-)	miR-654-3p/CCND1 axis	28
CircPLXNB2	↑	cell proliferation (+), migration (+), apoptosis (-)	regulated PLXNB2 expression	39
Circ-SFMBT2	↑	knockdown: cell apoptosis (+), proliferation (-), migration (-), invasion (-), glycolysis (-)	miR-582-3p/ZBTB20 axis	29
CircPTK2	↑	knockdown: cell apoptosis (+), cycle arrest (+), proliferation (-), glycolysis (-)	miR-582-3p/ALG3 axis	30
CircPTK2	↑	cell proliferation (-), apoptosis (+)	miR-330-5p/FOXM1 axis	31
Circ_KCNQ5	↑	knockdown: cell apoptosis (+), proliferation (-)	miR-622/RAB10	32
Circ_0005774	↑	knockdown: cell apoptosis (+), proliferation (-)	miR-192-5p/ULK1 axis	33
Circ_0094100	↑	knockdown: cell apoptosis (+), viability (-), cycle (-)	miR-217/ATP1B1 axis	34
CircRNA-DLEU2	↑	cell proliferation (+), apoptosis (-)	miR-496	35
Circ_POLA2	↑	cell proliferation (+)	miR-34a	36
Circ-ATAD1	↑	cell proliferation (+)	miR-34b	37
Hsa_circ_0079480	↑	reduction: cell apoptosis (+), growth (-)	miR-654-3p/HDGF axis	38
Circ_DLEU2	↑	knockdown: cell proliferation (-)	miR-582-5p	43
CircRNF220	↑	knockdown: cell apoptosis (+), proliferation (-), cycle progression (-), invasion (-), glycolytic metabolism (-)	miR-330-5p	44
CircRNF13	↑	downregulation: cell apoptosis (+), proliferation (-), migration (-), invasion (-)	miRNA-1224-5p	45
CircSH3BGRL3	↑	cell proliferation (+), DAU-resistance (+)	miR-375	46
CircNPM1	↑	silencing: cell colony formation (-), migration (-), invasion (-), Adriamycin (ADM) resistance (-), apoptosis (+), cycle arrest (+)	miR-345-5p/FZD5 axis	47
Circ_PAN3	↑	Doxorubicin (ADM)-resistant	miR-153-5p/miR-183-5p-XIAP	48
Circ_0001187	↓, newly diagnosed (ND) AML patients; ↑, patients HCR	knockdown: cell proliferation (+), apoptosis (-)	miR-499a-5p/RNF113A/METTL3	49
CircCRKL	↓	upregulation: cell proliferation (-), colony-forming ability (-)	miR-196a-5p/miR-196b-5p/p27 axis	50
Circ_0040823	↓	upregulation: cell apoptosis (+), cycle arrest (+), proliferation (-), growth of xenograft tumors *in vivo* (-)	miR-516b/PTEN	51
Hsa_circ_0121582	↓	upregulation: cell proliferation (-)	miR-224	52
Hsa_circ_0000370	↑, (FLT3-ITD+ AML)	tumor progression	miR-1299	53
CircMYBL2	↑	knockdown: cell proliferation (-), cytoactivity of inhibitor-resistant FLT3-ITD+ cell (-)	enhanced polypyrimidine bundle-binding protein 1 (PTBP1) binding of FLT3 messenger RNA	54
CircAF4	↑	knockdown: cell apoptosis (+)	miR-128-3p/MLL-AF4 axis	55
CircBCL11B	↑	knockdown: cell death (+), proliferation (-)	not covered	56
Circ-HIPK2	↓	cell proliferation (-), differentiation (-)	miR-124-3p	57

↑, high levels; ↓, low levels; (+), facilitating effect, (-) inhibiting effect.

**Table 2:** Overview of the potential effects of circRANS in CML.

CircRNA	Expression	Function	miRNAs, potential mechanism	Reference
Circ_BA9.3	↑	TKI resistance (+)	upregulated of the ABL1 and BCR-ABL1 protein	60
F-circBA1	↑	knockdown: cell proliferation (-)	miR-148-3p	63
Circ_SIRT1	↑	imatinib resistance (+),	regulated EIF4A3-mediated transcription of ATG12	65
Circ_0009910	↑	imatinib resistance (+), knockdown: cell apoptosis (+), proliferation (-), autophagy (-)	miR-34a-5p	66
CircCRKL	↑	imatinib resistance (+)	miR-877-5p	67
Circ_0080145	↑	imatinib resistance (+)	miR-203/ABL1	61
Circ_0051886	↑	imatinib resistance (+)	miR-637/ABL1	61
Circ_0080145	↑	imatinib resistance (+), silencing: imatinib resistance (-), cell growth (-), glycolysis (-), apoptosis (+)	miR-326/PPFIA1 axis	68
Hsa_circ_0080145	↑	knockdown: cell proliferation (-)	miR-29b	69
CircHIPK3	↑	poor outcome	miR-124	70
Circ_100053	↑	imatinib resistance (+)	not covered	71
Hsa_circ_0058493	↑	imatinib resistance (+)	miR-548b-3p	72

↑, high levels; ↓, low levels; (+), facilitating effect, (-) inhibiting effect.

**Table 3:** Overview of the potential effects of circRANS in ALL.

CircRNA	Expression	Function	miRNAs, potential mechanism	Reference
Circ_0008012	↑	knockdown: cell apoptosis (+), proliferation (-)	phosphorylated IκB and activated NF- κB:p65:p300 compound	75
Circ_0000094	↓	upregulation: cell proliferation (-), migration (-), invasion (-); apoptosis (+)	miR-223-3p/FBW7 axis	76
Circ_0000745	↑	overexpression: cell proliferation (+), apoptosis (-)	miR-193b-3p	77
CircPRKCI	↑	silencing: cell apoptosis (+)	miR-20a-5p/SOX4 axis	78
Circ_0000745	↑	knockdown: cell cycle progression (-), glycolysis (-), apoptosis (+), ferroptosis (+)	miR-494-3p/NET1 axis	79
Circ-PRKDC	↑	silencing: cell autophagy (+), apoptosis (+), proliferation (-)	miR-653-5p/RELN---PI3K/AKT/mTOR	80
CircADD2	↓	overexpression: cell proliferation (-), apoptosis (+)	miR-149-5p	81
Circ-0000745	↑	overexpression: cell proliferation (+), apoptosis (-)	increased the activity of ERK1/2	82
CircPVT1	↑	overexpression: cell proliferation (+), migration (+), invasion (+)	miR-125b	83
CircPVT1	↑	knockdown: cell proliferation (-), apoptosis (+)	miR-30e/DLL4 pathway	84
CircPVT1	↑	knockdown: cell proliferation (-), apoptosis (+)	inhibited gene c-Myc and antiapoptotic Bcl-2 protein expression	85
CircFBXW7	↑	knockdown: cell viability (+), proliferation (+), dexamethasone sensitivity (+)	sustained MYC and NOTCH1 activation	86

↑, high levels; ↓, low levels; (+), facilitating effect, (-) inhibiting effect.

**Table 4:** Overview of the potential effects of circRANS in CLL.

CircRNA	Expression	Function	miRNAs, potential mechanism	Reference
Circ-RPL15	↑	After RNAi of circ-RPL15: cell viability (-)	miR-146b-3p/RAS/RAF 1/MEK/ERK	90
CircTET2	↑	Overexpression: cell proliferation (+)	through mTORC1 signaling pathway	91
Circ_0002078	↑	Cell proliferation (+), metastasis (+)	miR-185-3p/TCF7L1 axis	92
CircRIC8B	↑	Poor prognosis; knockdown: cell proliferation (-), lipid accumulation (-)	miR-199b-5p/LPL axis	93
Circ-CBFB	↑	Knockdown: cell apoptosis (+), proliferation (-), arrested cell cycle progression (-)	miR-607/FZD3/Wnt/β-catenin	94
Circ_0132266	↓	Downregulation: cell proliferation (+), apoptosis (-)	miR-337- 3p/PML axis	95
CircZNF91	↑	Silencing: cell proliferation (-), apoptosis (+), cycle arrest (+)	miR-1283/WEE1 axis	96
Mc-COX2	↑	Decrease: cell apoptosis (+)	acted as the competing endogenous RNA	97

↑, high levels; ↓, low levels; (+), facilitating effect, (-) inhibiting effect.

**Table 5:** Overview of the potential effects of circRANS in Lymphoma.

Cancer type	CircRNA	Expression	Function	miRNAs, potential mechanism	Reference
DLBCL	CircPCBP2	↑	knockdown: cell stemness (-), CHOP sensitivity (+)	miR-33a/b/PD-L1 axis	100
	Circ_0003645	↑	knockdown: cell apoptosis (+), viability (-), cycle (-), glycolysis (-)	miR-335-5p/NFIB axis	101
	Circ_OTUD7A	↑	knockdown: cell cycle arrest (+), apoptosis (+), proliferation (-), metastasis (-)	miR-431-5p/FOXP1 axis	102
	CircSPEF2	↓	overexpression: cell apoptosis (+), proliferative activity (-)	miR-16-5p/BACH2 axis	103
	CircZNF609	↑	downregulation: cell proliferation (-)	miR-153	104
	Circ-APC (hsa_circ_0127621)	↓	ectopic expression: cell proliferation (-)	miR-888	105
	CircCFL1	↑	cell proliferation (+), migration (+)	miR-107	106
EBV + DLBCL	CircEAF2	↓	overexpression: cell apoptosis (+), epirubicin sensitivity (+)	miR-BART19-3p/APC/β-catenin axis	107
MCL	CircCTNNA1	↑	upregulation: poor survival; cell proliferation (+)	miR-34a	108
	CircCDYL	↑	knockdown: cell proliferation (-)	identified a circCDYL-micro (mi)RNA-mRNA/long non-coding (lnc)RNA network	109
NKTCL	CircADARB1	↑	knockdown: cell proliferation (-)	miR-214-3p	110
	CircKIF4A	↑	silencing: glucose uptake (-), lactate production (-)	miR-1231	111
T-LBL	Circ-LAMP1	↑	cell proliferation (+)	miR-615-5p/DDR2	112

↑, high levels; ↓, low levels; (+), facilitating effect, (-) inhibiting effect.

**Table 6:** Overview of the potential effects of circRANS in MM.

CircRNA	Expression	Function	miRNAs, potential mechanisms	Reference
Circ-CDYL	↑	depletion: cell growth (-); knockdown: growth *in vivo* (-)	miR-1180	117
Circ-MYBL2	↓	cell viability (-), DNA synthesis (-), cell cycle progression (-)	facilitated the binding of Cyclin F to MYBL2	118
Circ_0007841	↑	cell proliferation (+), cell cycle (+), motility (+), apoptosis (-)	miR-338-3p	119
CircXPO1	↑	silencing: cell G1 phase arrest, proliferation (-), apoptosis (+)	miR-495-3p/DDIT4 axis	120
Hsa_circ_0087776	↓	provide a basis for diagnosis	not covered	121
Circ-PTK2	↑	poor survival	MAPK and VEGF signaling pathways	122
Circ-RNF217	↑	poor survival		122
Circ-AFF2	↓	good survival		122
Circ_0000190	↓	high expression: better PFS and OS	miR-767-5p	123
Circ_0026652	↑	knockdown: BTZ sensitivity (+)	miR‑608‑mediated Wnt/β‑catenin	124
Circ_0001821	↑	poor OS; cell proliferation (+), arrested apoptosis (+)	not covered	125
CircMYC	↑	poor prognosis	not covered	126
Circ_0007841	↑	reflect the response and survival benefits to BTZ-based regimen in MM patients.	not covered	127
Circ_0000142	↑	overexpression: cell proliferation (+), migration (+), invasion (+), apoptosis (-)	miR-610/AKT3 axis	129
CircBUB1B_544aa	↑	chromosomal instability; poor outcome	contained the kinase catalytic center of BUB1B; interfere the MM microenvironmental cells	130
CircCHEK1_246aa	↑	chromosomal instability; cell invasive (+), osteoclast differentiation (+)	encoded and was translated to the CHEK1 kinase catalytic center	131
Circ-ATP10A	↑	MM angiogenesis (+)	miR-6758-3p/hsa-miR-3977/hsa-miR-6804-3p/hsa-miR-1266-3p/hsa-miR-3620-3p	132
CircHNRNPU	↑	adverse outcomes	interfered with various cells in the bone marrow microenvironment	133
Circ-CACNG2	↑	cardiomyocyte viability (-), cell apoptosis (+)	miR-197-3p/caspase3 axis	134
Circ-G042080	↑	MM-related myocardial damage	miRNA/TLR4 axis	135
CircPSAP	↑	depletion: cell apoptosis (+), BTZ sensitivity (+), proliferation (-)	miR-331-3p	136
Circ-CCT3	↑	silencing: BTZ sensitivity (+)	miR-223-3p/BRD4	137
CircRERE	↑	downregulation: BTZ resistance (-)	miR-152-3p/CD47 axis	138
Hsa_circ_0007841	↑	doxorubicin resistance (+)	upregulated the ABCG2 messenger RNA and protein level	139
CircITCH	↓	upregulation: BTZ sensitivity (+)	miR-615-3p/PRKCD	140
Circ_0005615	↑	knockdown: cell apoptosis (+), BTZ resistance (-), proliferation (-)	miR-185-5p/IRF4	141
Hsa_circ_0007841	↑	associated with disease prognosis、chromosomal aberrations	not covered	128
Circ_0007841	↑	knockdown: cell proliferation (-), chemoresistance (-), metastasis (-); apoptosis (+)	miR-129-5p/JAG1 axis	142
Circ_0000190	↓	cell apoptosis (+), viability (-), proliferation (-)	miR-767-5p	143
Circ-PTK2	↑	cell proliferation (+), migration (+), apoptosis (-)	miR-638/MEK&ERK、WNT&β-catenin	144
Circ_0058058	↑	*in vivo*, silencing: proliferation (-); *in vitro*, knockdown: apoptosis (+)	miR-338-3p	145
Circ_0003489	↑	cell proliferation (-), apoptosis (+)	miR-433-3p/PBX3 axis	146
CircKCNQ5	↑	knockdown: cell proliferation (-), migration (-), invasion (-), glycolysis (-), apoptosis (+)	miR-335-5p	147
Circ_SEC61A1	↑	silencing: cell proliferation (-), metastasis (-), apoptosis (+)	miR-660-5p/CDK6	148
Circ_0005615	↑	downregulation: cell proliferation (-), cycle progression (-), apoptosis (+)	miR-331-3p	149
CircATIC	↑	cell proliferation (+), migration (+), invasion (+), glycolysis (+), apoptosis (-)	miR-324-5p/HGF	150
Hsa_circ_0003489	↑	knockdown: cell apoptosis (+), viability (-), proliferation (-), autophagy (-)	miR-874-3p/HDAC1 axis	151
Circ_0111738	↓	overexpression: cell proliferation (-), migration (-), invasion (-), angiogenesis (-)	miR-1233-3p	152
Circ_0134426	↓	overexpression: cell growth (-), colony formation (-), migration (-)	miR-146b-3p	153
Circ_0069767	↑	overexpression: cell proliferation (-), migration (-), invasion (-), apoptosis (+)	miR-636	154

↑, high levels; ↓, low levels; (+), facilitating effect, (-) inhibiting effect.

## References

[1] Sanger HL, Klotz G, Riesner D, Gross HJ, Kleinschmidt AK (1976). Viroids are single-stranded covalently closed circular RNA molecules existing as highly base-paired rod-like structures. Proc Natl Acad Sci U S A.

[2] Nahand JS, Jamshidi S, Hamblin MR, Mahjoubin-Tehran M, Vosough M, Jamali M (2020). Circular RNAs: New Epigenetic Signatures in Viral Infections. Front Microbiol.

[3] Naeli P, Pourhanifeh MH, Karimzadeh MR, Shabaninejad Z, Movahedpour A, Tarrahimofrad H (2020). Circular RNAs and gastrointestinal cancers: Epigenetic regulators with a prognostic and therapeutic role. Crit Rev Oncol Hematol.

[4] Chen LL, Yang L (2015). Regulation of circRNA biogenesis. RNA Biol.

[5] Rybak-Wolf A, Stottmeister C, Glažar P, Jens M, Pino N, Giusti S (2015). Circular RNAs in the Mammalian Brain Are Highly Abundant, Conserved, and Dynamically Expressed. Mol Cell.

[6] Enuka Y, Lauriola M, Feldman ME, Sas-Chen A, Ulitsky I, Yarden Y (2016). Circular RNAs are long-lived and display only minimal early alterations in response to a growth factor. Nucleic Acids Res.

[7] Koh W, Pan W, Gawad C, Fan HC, Kerchner GA, Wyss-Coray T (2014). Noninvasive in vivo monitoring of tissue-specific global gene expression in humans. Proc Natl Acad Sci U S A.

[8] Li Z, Cheng Y, Wu F, Wu L, Cao H, Wang Q (2020). The emerging landscape of circular RNAs in immunity: breakthroughs and challenges. Biomark Res.

[9] Wang PL, Bao Y, Yee MC, Barrett SP, Hogan GJ, Olsen MN (2014). Circular RNA is expressed across the eukaryotic tree of life. PLoS One.

[10] Salzman J, Chen RE, Olsen MN, Wang PL, Brown PO (2013). Cell-type specific features of circular RNA expression. PLoS Genet.

[11] Xia S, Feng J, Lei L, Hu J, Xia L, Wang J (2017). Comprehensive characterization of tissue-specific circular RNAs in the human and mouse genomes. Brief Bioinform.

[12] Memczak S, Jens M, Elefsinioti A, Torti F, Krueger J, Rybak A (2013). Circular RNAs are a large class of animal RNAs with regulatory potency. Nature.

[13] Hansen TB, Jensen TI, Clausen BH, Bramsen JB, Finsen B, Damgaard CK (2013). Natural RNA circles function as efficient microRNA sponges. Nature.

[14] Meng S, Zhou H, Feng Z, Xu Z, Tang Y, Wu M (2019). Epigenetics in Neurodevelopment: Emerging Role of Circular RNA. Front Cell Neurosci.

[15] Cortés-López M, Gruner MR, Cooper DA, Gruner HN, Voda AI, van der Linden AM (2018). Global accumulation of circRNAs during aging in Caenorhabditis elegans. BMC Genomics.

[16] Chen W, Liu D, Li QZ, Zhu H (2019). The function of ncRNAs in rheumatic diseases. Epigenomics.

[17] Vo JN, Cieslik M, Zhang Y, Shukla S, Xiao L, Zhang Y (2019). The Landscape of Circular RNA in Cancer. Cell.

[18] Wu P, Mo Y, Peng M, Tang T, Zhong Y, Deng X (2020). Emerging role of tumor-related functional peptides encoded by lncRNA and circRNA. Mol Cancer.

[19] Chen B, Huang S (2018). Circular RNA: An emerging non-coding RNA as a regulator and biomarker in cancer. Cancer Lett.

[20] Yang H, Zhang H, Yang Y, Wang X, Deng T, Liu R (2020). Hypoxia induced exosomal circRNA promotes metastasis of Colorectal Cancer via targeting GEF-H1/RhoA axis. Theranostics.

[21] Hong W, Xue M, Jiang J, Zhang Y, Gao X (2020). Circular RNA circ-CPA4/ let-7 miRNA/PD-L1 axis regulates cell growth, stemness, drug resistance and immune evasion in non-small cell lung cancer (NSCLC). J Exp Clin Cancer Res.

[22] Kristensen LS, Hansen TB, Venø MT, Kjems J (2018). Circular RNAs in cancer: opportunities and challenges in the field. Oncogene.

[23] Bonizzato A, Gaffo E, Te Kronnie G, Bortoluzzi S (2016). CircRNAs in hematopoiesis and hematological malignancies. Blood Cancer J.

[24] Rahmati A, Mafi A, Soleymani F, Babaei Aghdam Z, Masihipour N, Ghezelbash B (2023). Circular RNAs: pivotal role in the leukemogenesis and novel indicators for the diagnosis and prognosis of acute myeloid leukemia. Front Oncol.

[25] Hu L, Zheng B, Yang Y, Chen C, Hu M (2023). Construction of circRNA-miRNA-mRNA Network Reveal Functional circRNAs and Key Genes in Acute Myeloid Leukemia. Int J Gen Med.

[26] Du A, Yang Q, Sun X, Zhao Q (2023). Exosomal circRNA-001264 promotes AML immunosuppression through induction of M2-like macrophages and PD-L1 overexpression. Int Immunopharmacol.

[27] Long F, Lin Z, Long Q, Lu Z, Zhu K, Zhao M (2023). CircZBTB46 Protects Acute Myeloid Leukemia Cells from Ferroptotic Cell Death by Upregulating SCD. Cancers (Basel).

[28] Wang J, Wu C, Zhou W (2023). CircPLXNB2 regulates acute myeloid leukemia progression through miR-654-3p/CCND1 axis. Hematology.

[29] Chang W, Shang Z, Ming X, Wu J, Xiao Y (2022). Circ-SFMBT2 facilitates the malignant growth of acute myeloid leukemia cells by modulating miR-582-3p/ZBTB20 pathway. Histol Histopathol.

[30] Shang Z, Ming X, Wu J, Liu W, Xiao Y (2022). CircPTK2 promotes cell viability, cell cycle process, and glycolysis and inhibits cell apoptosis in acute myeloid leukemia by regulating miR-582-3p/ALG3 axis. Expert Rev Hematol.

[31] Yi L, Zhou L, Luo J, Yang Q (2021). Circ-PTK2 promotes the proliferation and suppressed the apoptosis of acute myeloid leukemia cells through targeting miR-330-5p/FOXM1 axis. Blood Cells Mol Dis.

[32] Hu X, Yin J, He R, Chao R, Zhu S (2022). Circ_KCNQ5 participates in the progression of childhood acute myeloid leukemia by enhancing the expression of RAB10 via binding to miR-622. Hematology.

[33] Li Q, Luan Q, Zhu H, Zhao Y, Ji J, Wu F (2021). Circular RNA circ_0005774 contributes to proliferation and suppresses apoptosis of acute myeloid leukemia cells via circ_0005774/miR-192-5p/ULK1 ceRNA pathway. Biochem Biophys Res Commun.

[34] Cao J, Huang S, Li X (2022). Rapamycin inhibits the progression of human acute myeloid leukemia by regulating the circ_0094100/miR-217/ATP1B1 axis. Exp Hematol.

[35] Wu DM, Wen X, Han XR, Wang S, Wang YJ, Shen M (2018). Role of Circular RNA DLEU2 in Human Acute Myeloid Leukemia. Mol Cell Biol.

[36] Li H, Bi K, Feng S, Wang Y, Zhu C (2021). CircRNA circ_POLA2 is Upregulated in Acute Myeloid Leukemia (AML) and Promotes Cell Proliferation by Suppressing the Production of Mature miR-34a. Cancer Manag Res.

[37] Wu Y, Gao B, Qi X, Bai L, Li B, Bao H (2021). Circular RNA ATAD1 is upregulated in acute myeloid leukemia and promotes cancer cell proliferation by downregulating miR-34b via promoter methylation. Oncol Lett.

[38] Hu Q, Gu Y, Chen S, Tian Y, Yang S (2020). Hsa_circ_0079480 promotes tumor progression in acute myeloid leukemia via miR-654-3p/HDGF axis. Aging (Albany NY).

[39] Lin L, Wang Y, Bian S, Sun L, Guo Z, Kong D (2021). A circular RNA derived from PLXNB2 as a valuable predictor of the prognosis of patients with acute myeloid leukaemia. J Transl Med.

[40] Wei W, Pan J, Wang J, Mao S, Qian Y, Lin X (2023). circSLC25A13 acts as a ceRNA to regulate AML progression via miR-616-3p/ADCY2 axis. Mol Carcinog.

[41] Ping L, Jian-Jun C, Chu-Shu L, Guang-Hua L, Ming Z (2019). Silencing of circ_0009910 inhibits acute myeloid leukemia cell growth through increasing miR-20a-5p. Blood Cells Mol Dis.

[42] Liu X, Liu X, Cai M, Luo A, He Y, Liu S (2021). CircRNF220, not its linear cognate gene RNF220, regulates cell growth and is associated with relapse in pediatric acute myeloid leukemia. Mol Cancer.

[43] Dong S, Zhong H, Li L (2023). Circ_DLEU2 knockdown represses cell proliferation, migration and invasion, and induces cell apoptosis through the miR-582-5p/COX2 pathway in acute myeloid leukemia. Histol Histopathol.

[44] Zhang Z, Lin S, Yin J, Yu W, Xu C (2022). CircRNF220 plays a pathogenic role to facilitate cell progression of AML in vitro via sponging miR-330-5p to induce upregulation of SOX4. Histol Histopathol.

[45] Zhang R, Li Y, Wang H, Zhu K, Zhang G (2020). The Regulation of circRNA RNF13/miRNA-1224-5p Axis Promotes the Malignant Evolution in Acute Myeloid Leukemia. Biomed Res Int.

[46] Yang X, Wang Y, Rong S, An J, Lan X, Yin B (2023). Gene SH3BGRL3 regulates acute myeloid leukemia progression through circRNA_0010984 based on competitive endogenous RNA mechanism. Front Cell Dev Biol.

[47] Ding J, Zhang X, Xue J, Fang L, Ban C, Song B (2021). CircNPM1 strengthens Adriamycin resistance in acute myeloid leukemia by mediating the miR-345-5p/FZD5 pathway. Cent Eur J Immunol.

[48] Shang J, Chen WM, Wang ZH, Wei TN, Chen ZZ, Wu WB (2019). CircPAN3 mediates drug resistance in acute myeloid leukemia through the miR-153-5p/miR-183-5p-XIAP axis. Exp Hematol.

[49] Yang X, Han F, Hu X, Li G, Wu H, Can C (2023). EIF4A3-induced Circ_0001187 facilitates AML suppression through promoting ubiquitin-proteasomal degradation of METTL3 and decreasing m6A modification level mediated by miR-499a-5p/RNF113A pathway. Biomark Res.

[50] Liu W, Cheng F (2021). Circular RNA circCRKL inhibits the proliferation of acute myeloid leukemia cells via the miR-196a-5p/miR-196b-5p/p27 axis. Bioengineered.

[51] Wang N, Yang B, Jin J, He Y, Wu X, Yang Y (2022). Circular RNA circ_0040823 inhibits the proliferation of acute myeloid leukemia cells and induces apoptosis by regulating miR-516b/PTEN. J Gene Med.

[52] Chen JJ, Lei P, Zhou M (2020). hsa_circ_0121582 inhibits leukemia growth by dampening Wnt/β-catenin signaling. Clin Transl Oncol.

[53] Zhang L, Bu Z, Shen J, Shang L, Chen Y, Wang Y (2020). A novel circular RNA (hsa_circ_0000370) increases cell viability and inhibits apoptosis of FLT3-ITD-positive acute myeloid leukemia cells by regulating miR-1299 and S100A7A. Biomed Pharmacother.

[54] Sun YM, Wang WT, Zeng ZC, Chen TQ, Han C, Pan Q (2019). circMYBL2, a circRNA from MYBL2, regulates FLT3 translation by recruiting PTBP1 to promote FLT3-ITD AML progression. Blood.

[55] Huang W, Fang K, Chen TQ, Zeng ZC, Sun YM, Han C (2019). circRNA circAF4 functions as an oncogene to regulate MLL-AF4 fusion protein expression and inhibit MLL leukemia progression. J Hematol Oncol.

[56] Lux S, Blätte TJ, Gillissen B, Richter A, Cocciardi S, Skambraks S (2021). Deregulated expression of circular RNAs in acute myeloid leukemia. Blood Adv.

[57] Li S, Ma Y, Tan Y, Ma X, Zhao M, Chen B (2018). Profiling and functional analysis of circular RNAs in acute promyelocytic leukemia and their dynamic regulation during all-trans retinoic acid treatment. Cell Death Dis.

[58] Zhao X, Liu HQ, Wang LN, Yang L, Liu XL (2022). Current and emerging molecular and epigenetic disease entities in acute myeloid leukemia and a critical assessment of their therapeutic modalities. Semin Cancer Biol.

[59] Rudich A, Garzon R, Dorrance A (2022). Non-Coding RNAs Are Implicit in Chronic Myeloid Leukemia Therapy Resistance. Int J Mol Sci.

[60] Pan Y, Lou J, Wang H, An N, Chen H, Zhang Q (2018). CircBA9.3 supports the survival of leukaemic cells by up-regulating c-ABL1 or BCR-ABL1 protein levels. Blood Cells Mol Dis.

[61] Lu YH, Huang ZY (2021). Global identification of circular RNAs in imatinib (IM) resistance of chronic myeloid leukemia (CML) by modulating signaling pathways of circ_0080145/miR-203/ABL1 and circ 0051886/miR-637/ABL1. Mol Med.

[62] Ma Y, Zheng L, Gao Y, Zhang W, Zhang Q, Xu Y (2021). A Comprehensive Overview of circRNAs: Emerging Biomarkers and Potential Therapeutics in Gynecological Cancers. Front Cell Dev Biol.

[63] Tan Y, Huang Z, Wang X, Dai H, Jiang G, Feng W (2021). A novel fusion circular RNA F-circBA1 derived from the BCR-ABL fusion gene displayed an oncogenic role in chronic myeloid leukemia cells. Bioengineered.

[64] Xu C, Wang L, Fozouni P, Evjen G, Chandra V, Jiang J (2020). SIRT1 is downregulated by autophagy in senescence and ageing. Nat Cell Biol.

[65] Zhang R, Hao J, Yu H, Wang ZJ, Lan F, Peng Y (2024). circ_SIRT1 upregulates ATG12 to facilitate Imatinib resistance in CML through interacting with EIF4A3. Gene.

[66] Cao HX, Miao CF, Sang LN, Huang YM, Zhang R, Sun L (2020). Circ_0009910 promotes imatinib resistance through ULK1-induced autophagy by sponging miR-34a-5p in chronic myeloid leukemia. Life Sci.

[67] Wang J, Liang Y, Qin Y, Jiang G, Peng Y, Feng W (2022). circCRKL, a circRNA derived from CRKL, regulates BCR-ABL via sponging miR-877-5p to promote chronic myeloid leukemia cell proliferation. J Transl Med.

[68] Che H, Ding H, Jia X (2024). circ_0080145 Enhances Imatinib Resistance of Chronic Myeloid Leukemia by Regulating miR-326/PPFIA1 Axis. Cancer Biother Radiopharm.

[69] Liu J, Kong F, Lou S, Yang D, Gu L (2018). Global identification of circular RNAs in chronic myeloid leukemia reveals hsa_circ_0080145 regulates cell proliferation by sponging miR-29b. Biochem Biophys Res Commun.

[70] Feng XQ, Nie SM, Huang JX, Li TL, Zhou JJ, Wang W (2020). Circular RNA circHIPK3 serves as a prognostic marker to promote chronic myeloid leukemia progression. Neoplasma.

[71] Ping L, Jian-Jun C, Chu-Shu L, Guang-Hua L, Ming Z (2019). High circ_100053 predicts a poor outcome for chronic myeloid leukemia and is involved in imatinib resistance. Oncol Res.

[72] Zhong AN, Yin Y, Tang BJ, Chen L, Shen HW, Tan ZP (2021). CircRNA Microarray Profiling Reveals hsa_circ_0058493 as a Novel Biomarker for Imatinib-Resistant CML. Front Pharmacol.

[73] Kumar V, Jyotirmayee, Verma M (2023). Developing therapeutic approaches for chronic myeloid leukemia: a review. Mol Cell Biochem.

[74] Duffield AS, Mullighan CG, Borowitz MJ (2023). International Consensus Classification of acute lymphoblastic leukemia/lymphoma. Virchows Arch.

[75] Tang YL, Su JY, Luo JS, Zhang LD, Zheng LM, Liang C (2023). Gene Expression Network and Circ_0008012 Promote Progression in MLL/AF4 Positive Acute Lymphoblastic Leukemia. Recent Pat Anticancer Drug Discov.

[76] Hou Y, Sun J, Huang J, Yao F, Chen X, Zhu B (2021). Correction to: Circular RNA circRNA_0000094 sponges microRNA-223-3p and up-regulate F-box and WD repeat domain containing 7 to restrain T cell acute lymphoblastic leukemia progression. Hum Cell.

[77] Feng H, Li F, Tang P (2021). Circ_0000745 regulates NOTCH1-mediated cell proliferation and apoptosis in pediatric T-cell acute lymphoblastic leukemia through adsorbing miR-193b-3p. Hematology.

[78] Zheng Y, Niu B, Zhang W, Ru X, Gao Y, Li C (2021). Circular RNA circPRKCI contributes to malignant progression of T-cell acute lymphoblastic leukemia by modulating miR-20a-5p/SOX4 axis. Aging (Albany NY).

[79] Yang X, Li Y, Zhang Y, Liu J (2022). Circ_0000745 promotes acute lymphoblastic leukemia progression through mediating miR-494-3p/NET1 axis. Hematology.

[80] Ling Z, Fang ZG, Wu JY, Liu JJ (2021). The depletion of Circ-PRKDC enhances autophagy and apoptosis in T-cell acute lymphoblastic leukemia via microRNA-653-5p/Reelin mediation of the PI3K/AKT/mTOR signaling pathway. Kaohsiung J Med Sci.

[81] Zhu Y, Ma X, Zhang H, Wu Y, Kang M, Fang Y (2021). Mechanism of circADD2 as ceRNA in Childhood Acute Lymphoblastic Leukemia. Front Cell Dev Biol.

[82] Liu X, Zhou C, Li Y, Deng Y, Lu W, Li J (2020). Upregulation of circ-0000745 in acute lymphoblastic leukemia enhanced cell proliferation by activating ERK pathway. Gene.

[83] Jia Y, Hu H, Gu W (2021). Regulation Effects of Circular RNA CircPVT1 and miR-125b on NF-κB Signal Pathway in Childhood ALL. Clin Lab.

[84] Jia Y, Gu W (2021). Up-regulation of circPVT1 in T cell acute lymphoblastic leukemia promoted cell proliferation via miR-30e/DLL4 induced activating NOTCH signaling. Pathol Res Pract.

[85] Hu J, Han Q, Gu Y, Ma J, McGrath M, Qiao F (2018). Circular RNA PVT1 expression and its roles in acute lymphoblastic leukemia. Epigenomics.

[86] Buratin A, Borin C, Tretti Parenzan C, Dal Molin A, Orsi S, Binatti A (2023). CircFBXW7 in patients with T-cell ALL: depletion sustains MYC and NOTCH activation and leukemia cell viability. Exp Hematol Oncol.

[87] Li J, Peng T, Wang Y, Gan X, Zhang L (2023). Expression and Clinical Significance of circRNA Changes in Stored Erythrocytes During Platelet Transfusion in Acute Lymphoblastic Leukemia. Clin Lab.

[88] Alaggio R, Amador C, Anagnostopoulos I, Attygalle AD, Araujo IBO, Berti E (2022). The 5th edition of the World Health Organization Classification of Haematolymphoid Tumours: Lymphoid Neoplasms. Leukemia.

[89] Hampel PJ, Parikh SA (2022). Chronic lymphocytic leukemia treatment algorithm 2022. Blood Cancer J.

[90] Wu Z, Sun H, Liu W, Zhu H, Fu J, Yang C (2020). Circ-RPL15: a plasma circular RNA as novel oncogenic driver to promote progression of chronic lymphocytic leukemia. Leukemia.

[91] Wu Z, Zuo X, Zhang W, Li Y, Gui R, Leng J (2023). m6A-Modified circTET2 Interacting with HNRNPC Regulates Fatty Acid Oxidation to Promote the Proliferation of Chronic Lymphocytic Leukemia. Adv Sci (Weinh).

[92] Zhang X, Han Y, Hu X, Wang H, Tian Z, Zhang Y (2022). Competing endogenous RNA networks related to prognosis in chronic lymphocytic leukemia: comprehensive analyses and construction of a novel risk score model. Biomark Res.

[93] Wu Z, Gu D, Wang R, Zuo X, Zhu H, Wang L (2022). CircRIC8B regulates the lipid metabolism of chronic lymphocytic leukemia through miR199b-5p/LPL axis. Exp Hematol Oncol.

[94] Xia L, Wu L, Bao J, Li Q, Chen X, Xia H (2018). Circular RNA circ-CBFB promotes proliferation and inhibits apoptosis in chronic lymphocytic leukemia through regulating miR-607/FZD3/Wnt/β-catenin pathway. Biochem Biophys Res Commun.

[95] Wu W, Wu Z, Xia Y, Qin S, Li Y, Wu J (2019). Downregulation of circ_0132266 in chronic lymphocytic leukemia promoted cell viability through miR-337-3p/PML axis. Aging (Albany NY).

[96] Li S, Chen J, Fan Y, Xu X, Xiong M, Qi Y (2022). circZNF91 Promotes the Malignant Phenotype of Chronic Lymphocytic Leukemia Cells by Targeting the miR-1283/WEE1 Axis. Biomed Res Int.

[97] Wu Z, Sun H, Wang C, Liu W, Liu M, Zhu Y (2020). Mitochondrial Genome-Derived circRNA mc-COX2 Functions as an Oncogene in Chronic Lymphocytic Leukemia. Mol Ther Nucleic Acids.

[98] Hallek M, Al-Sawaf O (2021). Chronic lymphocytic leukemia: 2022 update on diagnostic and therapeutic procedures. Am J Hematol.

[99] Jiang M, Bennani NN, Feldman AL (2017). Lymphoma classification update: T-cell lymphomas, Hodgkin lymphomas, and histiocytic/dendritic cell neoplasms. Expert Rev Hematol.

[100] Dong L, Huang J, Gao X, Du J, Wang Y, Zhao L (2022). CircPCBP2 promotes the stemness and chemoresistance of DLBCL via targeting miR-33a/b to disinhibit PD-L1. Cancer Sci.

[101] Lin D, Wang Y, Lei L, Lin C (2022). Circ_0003645 serves as miR-335-5p sponge to promote the biological process of diffuse large B-cell lymphoma by upregulating NFIB. Autoimmunity.

[102] Liu W, Lei L, Liu X, Ye S (2021). CircRNA_OTUD7A upregulates FOXP1 expression to facilitate the progression of diffuse large B-cell lymphoma via acting as a sponge of miR-431-5p. Genes Genomics.

[103] Zhou J, Xu M, Chen Z, Huang L, Wu Z, Huang Z (2023). circ_SPEF2 Regulates the Balance of Treg Cells by Regulating miR-16-5p/BACH2 in Lymphoma and Participates in the Immune Response. Tissue Eng Regen Med.

[104] Yang CS, Lou Y, Ke QP, Xu XJ, Zhang Y (2022). [Mechanism of circZNF609 targeting miR-153 to regulate the proliferation and apoptosis of diffuse large B-cell lymphoma]. Zhonghua Zhong Liu Za Zhi.

[105] Hu Y, Zhao Y, Shi C, Ren P, Wei B, Guo Y (2019). A circular RNA from APC inhibits the proliferation of diffuse large B-cell lymphoma by inactivating Wnt/β-catenin signaling via interacting with TET1 and miR-888. Aging (Albany NY).

[106] Chen X, Xie X, Zhou W (2020). CircCFL1/MiR-107 Axis Targeting HMGB1 Promotes the Malignant Progression of Diffuse Large B-Cell Lymphoma Tumors. Cancer Manag Res.

[107] Zhao CX, Yan ZX, Wen JJ, Fu D, Xu PP, Wang L (2021). CircEAF2 counteracts Epstein-Barr virus-positive diffuse large B-cell lymphoma progression via miR-BART19-3p/APC/β-catenin axis. Mol Cancer.

[108] Lu C, Song C, Han Q, Jing X (2022). CircCTNNA1 is Upregulated in Mantle Cell Lymphoma and Predicts Poor Survival by Sponging miR-34a to Increase Cell Proliferation. Mediterr J Hematol Infect Dis.

[109] Mei M, Wang Y, Wang Q, Liu Y, Song W, Zhang M (2019). CircCDYL Serves as a New Biomarker in Mantle Cell Lymphoma and Promotes Cell Proliferation. Cancer Manag Res.

[110] Mei M, Wang Y, Song W, Li Z, Wang Q, Li J (2021). CircADARB1 serves as a new biomarker in natural killer T-cell lymphoma and a potential regulator of p-Stat3. Cancer Cell Int.

[111] He R, Wen W, Fu B, Zhu R, Chen G, Bai S (2022). CircKIF4A Is a Prognostic Factor and Modulator of Natural Killer/T-Cell Lymphoma Progression. Cancers (Basel).

[112] Deng L, Liu G, Zheng C, Zhang L, Kang Y, Yang F (2019). Circ-LAMP1 promotes T-cell lymphoblastic lymphoma progression via acting as a ceRNA for miR-615-5p to regulate DDR2 expression. Gene.

[113] van de Donk N, Pawlyn C, Yong KL (2021). Multiple myeloma. Lancet.

[114] Cowan AJ, Green DJ, Kwok M, Lee S, Coffey DG, Holmberg LA (2022). Diagnosis and Management of Multiple Myeloma: A Review. Jama.

[115] Mitsiades CS (2021). Biological and Translational Considerations regarding the Recent Therapeutic Successes and Upcoming Challenges for Multiple Myeloma. Cold Spring Harb Perspect Med.

[116] Mirazimi Y, Aghayan AH, Keshtkar A, Mottaghizadeh Jazi M, Davoudian A, Rafiee M (2023). CircRNAs in diagnosis, prognosis, and clinicopathological features of multiple myeloma; a systematic review and meta-analysis. Cancer Cell Int.

[117] Chen F, Wang X, Fu S, Wang S, Fu Y, Zhang J (2020). Circular RNA circ-CDYL sponges miR-1180 to elevate yes-associated protein in multiple myeloma. Exp Biol Med (Maywood).

[118] Yu S, Ai L, Wei W, Pan J (2021). circRNA circ-MYBL2 is a novel tumor suppressor and potential biomarker in multiple myeloma. Hum Cell.

[119] Wang Y, Lin Q, Song C, Ma R, Li X (2020). Circ_0007841 promotes the progression of multiple myeloma through targeting miR-338-3p/BRD4 signaling cascade. Cancer Cell Int.

[120] Li F, Liu J, Miao J, Hong F, Liu R, Lv Y (2024). Circular RNA circXPO1 Promotes Multiple Myeloma Progression by Regulating miR-495-3p/DNA Damage-Induced Transcription 4 Axis. DNA Cell Biol.

[121] Gong X, Lu X, Cao J, Liu H, Chen H, Bao F (2021). Serum hsa_circ_0087776 as a new oncologic marker for the joint diagnosis of multiple myeloma. Bioengineered.

[122] Zhou F, Wang D, Wei W, Chen H, Shi H, Zhou N (2020). Comprehensive profiling of circular RNA expressions reveals potential diagnostic and prognostic biomarkers in multiple myeloma. BMC Cancer.

[123] Xiang Y, Xu X, Yang B, Wu Z, Jiang R, Xie Y (2022). Circular RNA_0000190 and its target microRNA-767-5p are dysregulated, and they are related to risk stratification as well as prognosis in multiple myeloma patients. Ir J Med Sci.

[124] Li L, Liu J, Du J, Jiang H, He H, Lu J (2022). Dysregulated circular RNAs are closely linked to multiple myeloma prognosis, with circ_0026652 predicting bortezomib-based treatment response and survival via the microRNA-608-mediated Wnt/β-catenin pathway. Oncol Rep.

[125] Liu L, Zhang F, Li J (2021). CircRNA circ_0001821 predicts an unfavorable prognosis and promotes the proliferation of multiple myeloma. Hematology.

[126] Luo Y, Gui R (2020). Circulating Exosomal CircMYC Is Associated with Recurrence and Bortezomib Resistance in Patients with Multiple Myeloma. Turk J Haematol.

[127] Guo Y, Feng X, Wang Z, Zhang R, Zheng K, Xu J (2024). The quantification of circular RNA 0007841 during induction therapy helps estimate the response and survival benefits to bortezomib-based regimen in multiple myeloma. Ir J Med Sci.

[128] Gao M, Li C, Xiao H, Dong H, Jiang S, Fu Y (2019). hsa_circ_0007841: A Novel Potential Biomarker and Drug Resistance for Multiple Myeloma. Front Oncol.

[129] Liu F, Wang YL, Wei JM, Huang ZD (2021). Upregulation of circ_0000142 promotes multiple myeloma progression by adsorbing miR-610 and upregulating AKT3 expression. J Biochem.

[130] Tang X, Guo M, Ding P, Deng Z, Ke M, Yuan Y (2021). BUB1B and circBUB1B_544aa aggravate multiple myeloma malignancy through evoking chromosomal instability. Signal Transduct Target Ther.

[131] Gu C, Wang W, Tang X, Xu T, Zhang Y, Guo M (2021). CHEK1 and circCHEK1_246aa evoke chromosomal instability and induce bone lesion formation in multiple myeloma. Mol Cancer.

[132] Yu M, Yu J, Zhang Y, Sun X, Sun R, Xia M (2022). A novel circRNA-miRNA-mRNA network revealed exosomal circ-ATP10A as a biomarker for multiple myeloma angiogenesis. Bioengineered.

[133] Tang X, Deng Z, Ding P, Qiang W, Lu Y, Gao S (2022). A novel protein encoded by circHNRNPU promotes multiple myeloma progression by regulating the bone marrow microenvironment and alternative splicing. J Exp Clin Cancer Res.

[134] Yu M, Ji L, Li S, Zhang Y, Sun X, Sun R (2022). Exosomal circ-CACNG2 promotes cardiomyocyte apoptosis in multiple myeloma via modulating miR-197-3p/caspase3 axis. Exp Cell Res.

[135] Sun R, Liu W, Zhao Y, Chen H, Wang Z, Zhang Y (2021). Exosomal circRNA as a novel potential therapeutic target for multiple myeloma-related myocardial damage. Cancer Cell Int.

[136] Ma H, Shen L, Yang H, Gong H, Du X (2022). Circular RNA circPSAP functions as an efficient miR-331-3p sponge to regulate proliferation, apoptosis and bortezomib sensitivity of human multiple myeloma cells by upregulating HDAC4. J Pharmacol Sci.

[137] Liu D, Wang Y, Li H, Peng S, Tan H, Huang Z (2022). Circular RNA circ-CCT3 promotes bortezomib resistance in multiple myeloma via modulating miR-223-3p/BRD4 axis. Anticancer Drugs.

[138] Fang W, Mu J, Yang Y, Liu L (2021). CircRERE confers the resistance of multiple myeloma to bortezomib depending on the regulation of CD47 by exerting the sponge effect on miR-152-3p. J Bone Oncol.

[139] Song Y, Hu N, Song X, Yang J (2020). Hsa_Circ_0007841 Enhances Multiple Myeloma Chemotherapy Resistance Through Upregulating ABCG2. Technol Cancer Res Treat.

[140] Liu J, Du F, Chen C, Li D, Chen Y, Xiao X (2020). CircRNA ITCH increases bortezomib sensitivity through regulating the miR-615-3p/PRKCD axis in multiple myeloma. Life Sci.

[141] Fu C, Wang J, Hu M, Zhou W (2022). Circ_0005615 contributes to the progression and Bortezomib resistance of multiple myeloma by sponging miR-185-5p and upregulating IRF4. Anticancer Drugs.

[142] Wang Y, Lin Q, Song C, Ma R, Li X (2020). Depletion of circ_0007841 inhibits multiple myeloma development and BTZ resistance via miR-129-5p/JAG1 axis. Cell Cycle.

[143] Feng Y, Zhang L, Wu J, Khadka B, Fang Z, Gu J (2019). CircRNA circ_0000190 inhibits the progression of multiple myeloma through modulating miR-767-5p/MAPK4 pathway. J Exp Clin Cancer Res.

[144] Zhou F, Wang D, Zhou N, Chen H, Shi H, Peng R (2021). Circular RNA Protein Tyrosine Kinase 2 Promotes Cell Proliferation, Migration and Suppresses Apoptosis via Activating MicroRNA-638 Mediated MEK/ERK, WNT/β-Catenin Signaling Pathways in Multiple Myeloma. Front Oncol.

[145] Xue L, Jia T, Zhu Y, Zhao L, Mao J (2021). Down-regulation of circ_0058058 suppresses proliferation, angiogenesis and metastasis in multiple myeloma through miR-338-3p/ATG14 pathway. J Orthop Surg Res.

[146] Yan T, Wang X, Zhou D, Qiu H, Zhang J, Yang W (2022). Circ_0003489 facilitates multiple myeloma progression by targeting miR-433-3p/PBX3 axis. Hematology.

[147] Li Y, Wang L, Zhang N, Xu Y (2023). CircKCNQ5 controls proliferation, migration, invasion, apoptosis, and glycolysis of multiple myeloma cells by modulating miR-335-5p/BRD4 axis. Histol Histopathol.

[148] Luo Z, Yin Y, Tan X, Liu K, Chao Z, Xia H (2022). Circ_SEC61A1 contributes to the progression of multiple myeloma cells via regulating miR-660-5p/CDK6 axis. Leuk Res.

[149] Zhang Q, Duan H, Yang W, Liu H, Tao X, Zhang Y (2023). Circ_0005615 restrains the progression of multiple myeloma through modulating miR-331-3p and IGF1R regulatory cascade. J Orthop Surg Res.

[150] Wu B, Wang F, Wang Y, Deng X, Wu W (2022). CircATIC Contributes to Multiple Myeloma Progression via miR-324-5p-Dependent Regulation of HGF. Biochem Genet.

[151] Tian FQ, Chen ZR, Zhu W, Tang MQ, Li JH, Zhang XC (2021). Inhibition of hsa_circ_0003489 shifts balance from autophagy to apoptosis and sensitizes multiple myeloma cells to bortezomib via miR-874-3p/HDAC1 axis. J Gene Med.

[152] Wang P, Zhang Y, Lin Q, Zhou J, Lv X, Song Y (2023). Hsa_circ_0111738 Inhibits Tumor Progression and Angiogenesis in Multiple Myeloma by Sponging miR-1233-3p to Regulate HIF-1 Signaling Pathway. Arch Med Res.

[153] Li M, Li J, Song Y (2023). Hsa_Circ_0134426 Attenuates the Malignant Biological Behaviors of Multiple Myeloma by Suppressing miR-146b-3p to Upregulate NDNF. Mol Biotechnol.

[154] Chen F, Wang X, Fu S, Wang S, Fu Y, Liu Z (2020). Effect of the Up-Regulation of Circular RNA Hsa_circ_0069767 Derived from C-KIT on the Biological Behavior of Multiple Myeloma Cells. Cancer Manag Res.

